# Thiolated Polymers in 3D Bioprinting: Control of Gelation

**DOI:** 10.1002/adma.73394

**Published:** 2026-05-22

**Authors:** Soheil Haddadzadegan, Flavia Laffleur, Andreas Bernkop‐Schnürch

**Affiliations:** ^1^ Center For Sustainable Materials (SusMat) School of Materials Science and Engineering Nanyang Technological University Singapore Singapore; ^2^ Department of Pharmaceutical Technology Institute of Pharmacy University of Innsbruck Innsbruck Austria

## Abstract

Thiolated polymers represent a versatile class of bioinks for extrusion‐based 3D bioprinting, combining cytocompatibility with tunable crosslinking chemistry and dynamic redox‐responsive behaviour. This review consolidates recent advances in thiomer chemistry, focusing on synthetic strategies that modulate thiol reactivity through pKa adjustment, neighboring‐group interactions, and redox control. Crosslinking mechanisms such as oxidative disulfide formation, thiol–ene, thiol‐yne, and thiol‐polyphenol reactions are compared in terms of their impact on gelation. External triggers, including small‐molecule and polymeric crosslinkers, light activation, oxidants, enzymatic systems, as well as hybrid dual‐stage systems, are discussed for their capacity to achieve controlled gelation and long‐term stability. A comprehensive printability framework links chemical design to performance metrics such as gel point, modulus build‐up rate, collapse angle, filament fusion index, fidelity ratio, and shear thresholds that maintain cell viability. Redox‐driven reversibility provides additional adaptability through self‐healing and stress‐relaxation mechanisms. Applications span soft tissue and cartilage regeneration, vascularized and multicellular constructs, hemostatic adhesives, and extracellular matrix–mimetic scaffolds for stem‐cell culture. These developments collectively establish design principles for balancing gelation kinetics, shape fidelity, and biological functionality in thiomer‐based bioinks.

## Graphical abstract

Thiolated polymers are established as programmable bioinks for 3D bioprinting, integrating versatile crosslinking chemistries with redox‐responsive control. This work demonstrates how molecular design and external triggers define gelation kinetics, printability windows, and structural fidelity, enabling stable, high‐resolution constructs and advancing applications in soft tissue engineering, vascular fabrication, and ECM‐mimetic scaffolds.

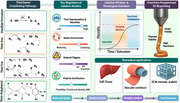

## 1 Introduction

Three‐dimensional (3D) bioprinting has rapidly emerged as a transformative technology in tissue engineering and regenerative medicine, enabling the fabrication of cell‐laden constructs with spatial precision and biomimetic architectures [1, 2, 3]. A critical requirement for successful bioprinting is the use of bioinks that can undergo rapid yet controllable gelation, ensuring high shape fidelity during printing while preserving cell viability and biological function [4, 5, 6]. Among the diverse classes of bioinks under investigation, thiolated polymers, commonly referred to as thiomers, have attracted significant attention due to their unique chemical versatility and biofunctional properties [7, 8]. Thiomers were first introduced in the late 1990s as a means to enhance the mucoadhesive properties of established polymers such as polyacrylates, hyaluronic acid, and chitosan through covalent attachment of thiol groups [9, 10]. The introduction of free thiols conferred the ability to form disulfide bonds both with cysteine‐rich substructures on biological surfaces and within polymeric networks themselves [11, 12, 13, 14, 15, 16, 17]. This innovation resulted in significant improvements in bioadhesion of printed constructs to the target tissue and established thiomers as a valuable platform for tissue engineering and regenerative medicine [18, 19]. Beyond adhesion, thiomers also mimic key features of the extracellular matrix (ECM), supporting cell anchorage and enabling tunable biodegradability through controlled degrees of thiolation [20, 21]. Early work demonstrated their capacity as ECM mimics for fibroblast culture [22], and since then, thiomer‐based systems have steadily advanced toward preclinical and clinical applications. With the advent of 3D bioprinting in the 2010s, thiomers were incorporated into bioinks, leveraging their intrinsic crosslinking capability and biocompatibility. In this context, the challenge of controlling gelation kinetics has become central. Gelation must be sufficiently rapid to stabilize printed filaments and preserve pore interconnectivity, which is essential for nutrient diffusion and oxygen transport [23]. At the same time, premature crosslinking can cause nozzle clogging or prevent smooth extrusion [24, 25, 26]. Achieving this delicate balance requires a precise understanding of the reactivity of thiol groups, their interactions with electron‐donating or electron‐withdrawing substituents, and the influence of the surrounding electrostatic and redox environment. Several chemistries have been harnessed to modulate thiomer gelation, including oxidative disulfide formation, thiol–ene and thiol‐yne click chemistry, and interactions with polyphenolic moieties. These processes can be further tuned by external triggers such as light, oxidizing agents, or co‐axial delivery of crosslinkers. Despite significant progress, no universal strategy has yet achieved complete control over gelation kinetics in a way that simultaneously optimizes printability, structural fidelity, and biological performance. In this review, we provide a comprehensive overview of thiolated polymers in the context of 3D bioprinting, with particular emphasis on the chemical mechanisms and external strategies available to regulate gelation times. We first discuss the evolution and properties of thiomers, followed by a detailed examination of their reactivity and crosslinking modes. We then highlight how these mechanisms can be fine‐tuned during printing and survey current applications in tissue engineering. Finally, we outline the key challenges that remain and discuss future directions for advancing thiomer‐based bioinks toward clinical translation.

## 2 Thiomers for 3D Bioprinting

A wide variety of thiomers is currently available for 3D bioprinting, and the number of newly developed thiomers continues to grow. These materials are typically synthesized by covalently introducing free thiol groups (─SH) onto well‐established natural or synthetic polymer backbones. Common polymers include polysaccharides such as hyaluronic acid, alginate, chitosan, heparin or chondroitin sulfate, proteins such as gelatin, collagen, or elastin, and synthetic polymers such as poly(meth)acrylates and polyethylene glycols (PEGs). The introduction of thiols can be achieved through a variety of chemical routes, including the covalent attachment of sulfhydryl ligands and hydroxy‐to‐thiol substitution reactions using thiourea or phosphorus pentasulfide [21]. The degree of thiolation typically ranges from 100–500 µmol thiol groups per gram of polymer, though higher levels exceeding 1000 µmol/g can be achieved with substitution reactions [27]. The scale‐up and large‐scale production of thiomers is relatively straightforward. In most cases, sulfhydryl ligands are covalently attached to the polymer backbone via click chemistry in aqueous media. Since manufacturers of polysaccharides and proteins already employ well‐established purification methods, these processes can be readily adapted for thiomers following the coupling reaction. However, a key bottleneck, common to all polysaccharides and proteins used in 3D bioprinting, is the lack of highly efficient, industrially suitable sterilization techniques.

Although many research groups still prefer to synthesize thiomers in‐house to tailor their properties to specific requirements, a growing range of commercially available thiolated polymers is now accessible for applications in tissue engineering and bioprinting. For instance, Advanced BioMatrix Inc. (USA) offers multiple thiolated biomacromolecules, including hyaluronic acid, gelatin, and heparin. Similarly, HAworks LLC. (USA) provides a broad range of thiolated polysaccharides, such as hyaluronic acid, alginate, chitosan, dextran, and chondroitin sulfate, available in different polymer chain lengths. Biopharma PEG Scientific Inc. (USA) specializes in thiolated PEGs with molecular weights ranging from 10 to 40 kDa. In Europe, Blafar Ltd. (Ireland) supplies thiolated hyaluronic acid and gelatin. Meanwhile, Xi'an Ruixi Biological Technology Co. (China) offers an extensive portfolio, including thiolated and S‐protected thiolated chitosans, thiolated chitosan–PEG conjugates, thiolated alginate, and thiolated hyaluronic acid. Additionally, CycloLab Cyclodextrin Research and Development Laboratory Ltd. (Hungary) provides various thiolated cyclodextrin derivatives.

Compared to other polymers used in bioprinting, thiomers represent a unique class of materials that mimic endogenous binding and crosslinking mechanisms by utilizing nature's key bridging structure, the disulfide bond. Thiomers are capable of self‐crosslinking, whereas most other bioprinting polymers, such as ene‐, yne‐, epoxy‐, or halide‐functionalized systems, require additional reactive groups to achieve crosslinking. Moreover, thiol groups exhibit strong adhesive properties toward cells and tissues by forming disulfide bonds with cysteine‐rich membrane proteins, such as keratins. Compared to other polymers used in bioprinting, thiomers also offer exceptional flexibility in the reactivity of their functional groups, as discussed in detail in the following section. In addition, a high degree of versatility is available in the choice of polymer backbone, ranging from biodegradable to non‐biodegradable systems, from anionic and non‐ionic to cationic materials, and from short to long chain lengths. For further guidance, the most important performance indicators for thiomers used in tissue engineering and bioprinting are summarized in Table 1.

**TABLE 1:** Performance indicators for thiomers used in tissue engineering and bioprinting.

Performance indicator	Additional information
Degree of thiolation (>250 µmol/g)	To achieve sufficient crosslinking, and thus adequate gelation and mechanical strength, the degree of thiolation should exceed 250 µmol per gram of polymer.
Sufficient thiol reactivity	Thiol groups must exhibit sufficiently high reactivity to ensure rapid gelation. However, excessively high reactivity may lead to premature gelation during printing and reduced storage stability due to unintended disulfide bond formation.
High safety profile	Ideally, thiolated polymers consist of endogenous components, as in hyaluronic acid–cysteine conjugates. Alternatively, all components should be generally recognized as safe (GRAS).
Biodegradability	Biodegradable backbones, such as polysaccharides and proteins, are preferred over non‐biodegradable materials like polymethacrylates and PEGs.
Additional functionalities	Features such as sustained release of active agents (e.g., growth factors or antibiotics) or bioresponsive behavior are often important selection criteria.

## 3 Reactivity of Thiomers

The reactivity of thiol groups is a critical parameter for controlling the gelation time of thiomers [28]. It depends on the electron density around the thiol group or disulfide bond. In case of free thiols, electron density is characterized by pKa that is governed by the electron‐withdrawing properties of the attached ligand [29, 30]. The stronger the electron‐withdrawing effect, the lower is the pKa. A lower pKa corresponds to a higher concentration of thiolate anions, which represent the reactive form of thiols, thereby increasing overall reactivity. The rate of thiol–disulfide exchange is influenced by the pKa of thiol groups, which can be tuned through the incorporation of electron‐withdrawing substituents. Table 2 lists various thiol ligands along with their corresponding pKa values. As a general rule, lower pKa values indicate higher thiol reactivity, which is crucial for tailoring the gelation kinetics of thiomer‐based hydrogels.

**TABLE 2:** Reactivity of representative thiol ligands for thiol/disulfide exchange reactions and oxidation; pKa values of thiol groups were calculated using “ADMET Predictor version 9.0.0.10”.

Ligand	Structure	pKa
4‐Mercaptobenzoic acid	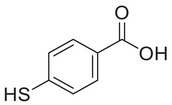	5.80
4‐Aminothiophenol	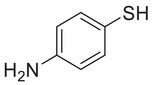	7.06
L‐Cysteine	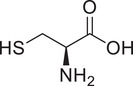	8.02
L‐Cysteine ethyl ester	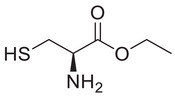	9.54
Homocysteine	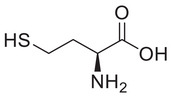	10.50

**FIGURE 1:**
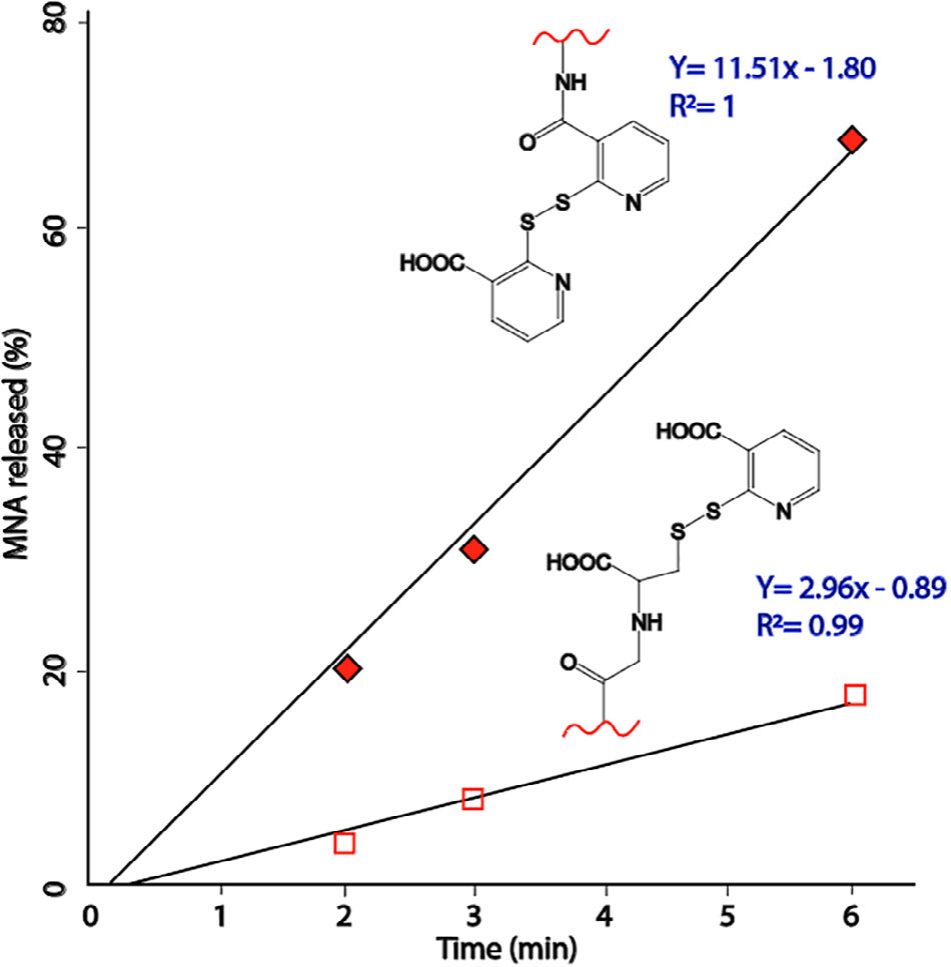
Reactivity of S‐protected chitosans with different electron‐withdrawing flanking groups, assessed by monitoring the release of mercaptonicotinic acid (MNA) in the presence of reduced glutathione, indicating the efficiency of thiol/disulfide exchange reactions, adapted and redrawn with permission from [37]. Copyright 2016, Elsevier.

The pKa of thiol groups is also influenced by the surrounding environment [31, 32]. For example, the thiol group of the cysteine residue in the active site of papain exhibits a significantly lower pKa of 4, compared to the typical value of around 8 [33]. This shift is due to the formation of a thiolate–imidazolium ion pair between the cysteine side chain and a nearby histidine residue [34]. Bermejo‐Velasco and co‐workers achieved dramatically faster gelation at physiological pH by introducing electron‐withdrawing groups, reducing the gelation time from approximately 10 h for hyaluronic‐acetyl‐cysteine to about 3.5 min for hyaluronic‐cysteine. In contrast, the least reactive conjugate, hyaluronic‐3‐mercaptopropionic acid, which has the highest pKa, failed to gel under the same conditions [35]. Similarly, the reactivity of disulfide bonds in thiol/disulfide exchange reactions is affected by the electron‐withdrawing properties of adjacent groups [36]. For instance, the pyridine ring in mercaptonicotinic acid exerts a strong electron‐withdrawing effect [37]. As a result, disulfide bonds flanked by two pyridine substructures exhibit significantly higher reactivity toward exchange with glutathione compared to those flanked by only one pyridine moiety, as illustrated in Figure 1.

In addition, charged groups neighboring free thiols and disulfides play a crucial role in disulfide bond formation and thiol/disulfide exchange reactions [36, 38]. Cationic neighboring groups accelerate disulfide bond formation when the thiols are flanked by anionic substructures, and vice versa. Conversely, when thiol groups or disulfide bonds are adjacent to groups with the same charge, electrostatic repulsion occurs, hindering disulfide bond formation and thiol/disulfide exchange reactions [39]. The influence of the electrostatic environment on thiol/disulfide exchange has, for example, been evaluated using cysteine flanked by two neutral groups, a neutral and a positively charged group, or two positively charged groups. The rate constants for reactions with the disulfide of the negatively charged Ellman's reagent were found to be 367, 3350, and 1 32 000 s^−^^1^·M^−^^1^, respectively [40]. Additionally, other parameters, such as the redox potential of thiol groups, can also significantly affect their reactivity [41]. Taking these parameters into consideration, the reactivity of thiomers and consequently gelation times can be adjusted. Highly reactive free thiols, however, tend to oxidize and/or to react with crosslinkers already before the printing process. Under inert conditions, they would be stable toward oxidation even in aqueous media, but as embedded cells need oxygen this approach cannot be applied for bioprinting. Consequently, less reactive free thiols or S‐protected thiols are favoured.

## 4 Crosslinking Reactions

**FIGURE 2:**
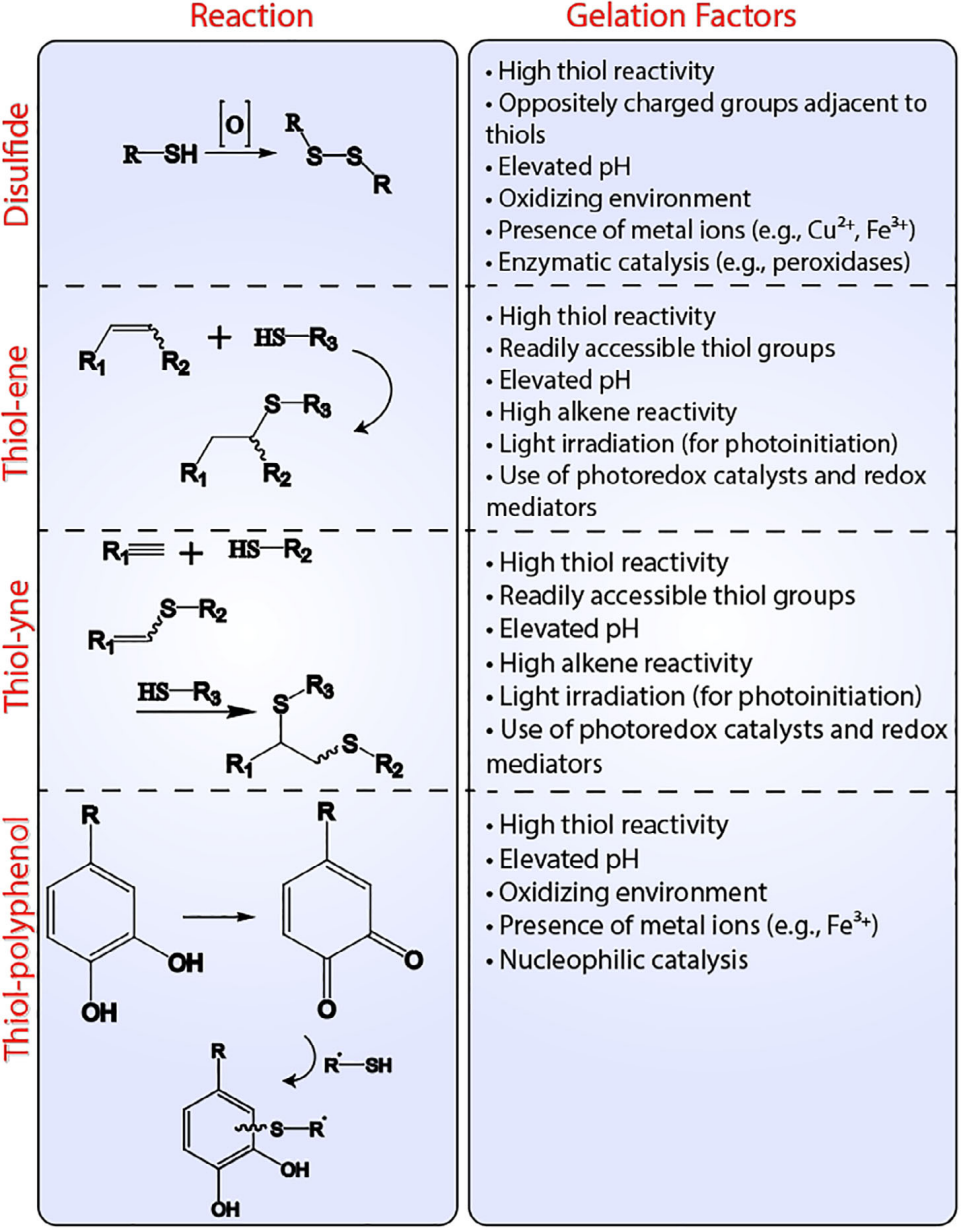
Thiol‐based crosslinking mechanisms governing hydrogel gelation in 3D bioprinting.

Thiomers are primarily crosslinked via disulfide bond formation, as well as through thiol–ene, thiol–yne, and thiol–polyphenol reactions. Other crosslinking mechanisms, such as thiol–epoxy or thiol–halide reactions, are of limited relevance for 3D bioprinting, mainly due to safety concerns. A detailed overview of the crosslinking of thiomers is provided by Summonte et al. [42]. Beyond the specific reaction pathways, thiomer gelation is also governed by a combination of intrinsic polymer properties and externally applied triggers. Intrinsic parameters such as thiol concentration, thiol pKa, and neighboring‐group effects directly influence the availability and reactivity of thiolate species, thereby modulating the rate and extent of covalent crosslinking. In parallel, several extrinsic factors, including the presence of crosslinkers, oxidizing agents, enzymatic activators, redox mediators, UV‐light exposure, and other chemical stimuli, serve as powerful tools to accelerate, initiate, or fine‐tune the gelation process. Together, these intrinsic and extrinsic elements define the overall gelation kinetics, mechanical performance, and functional behavior of thiomer‐based hydrogels. Figure 2 provides an overview of thiol‐based crosslinking mechanisms and the key factors governing hydrogel gelation in 3D bioprinting.

Given the diversity of thiol‐based crosslinking chemistries and their distinct implications for gelation control and printability, a comparative summary of representative studies is provided in Table 3 to guide rational bioink design.

**TABLE 3:** Comparative overview of thiol‐based crosslinking strategies in thiolated polymer bioinks: Mechanisms, representative studies, and bioprinting relevance.

Crosslinking mechanism	Polymer system	Trigger/Chemistry	Key outcome	Bioprinting relevance	Refs.
Disulfide oxidation	Thiolated polymers (general)	Oxygen‐mediated thiol oxidation	Spontaneous gelation; rate dependent on pH, thiol content	Limited control; requires fresh preparation before printing	[43, 44]
Thiol–disulfide exchange	S‐protected thiomers (e.g., HA)	Nucleophilic thiolate attack on disulfide bonds	Controlled activation via deprotection; oxygen‐independent	Enables on‐demand gelation; suitable for controlled systems	[12, 36]
Disulfide exchange via biological trigger	S‐protected HA	Endogenous thiols (e.g., glycoproteins, NAC)	Stable during storage; activated in biological environment	Ideal for in situ gelation and biomedical applications	[45]
Thiol–ene reaction	Poly(N‐isopropylacrylamide)	Base‐catalyzed thiolate addition to vinyl groups	Dual gelation (physical + chemical)	Tunable gelation kinetics and mechanical properties	[46]
Thiol–ene reaction	Acrylated polymers + 4‐arm thiol crosslinker	Nucleophilic addition	Efficient network formation but requires high pH	Limited by toxicity of crosslinker	[47, 48]
Thiol–ene reaction	Thiolated thermo‐responsive polymer + PEG diacrylate	Michael addition	Eliminates toxic crosslinker; improved biocompatibility	Better suited for bioink development	[49]
Thiol–ene reaction	HA‐SH + methacrylated HA	Photoinitiator + blue light	Injectable hydrogel formed in situ	Enables spatial control; minimally invasive applications	[50]
Thiol–norbornene reaction	PEG‐based systems	Radical‐mediated step‐growth polymerization	Fast kinetics; oxygen‐insensitive; high efficiency	Ideal for rapid bioprinting and photopatterning	[51]
Thiol–polyphenol reaction	HA–catechol + PEG‐SH	Michael‐type addition	Strong adhesion and self‐healing	Suitable for tissue adhesive bioinks	[52]
Thiol–polyphenol reaction	Catechol‐chitosan + thiolated poloxamer	Controlled thiol–catechol reaction	Tunable gelation speed; strong adhesion	Promising for injectable and printable hydrogels	[53]
Thiol‐polyphenol reaction	Thiol–catechol polymers	Sodium periodate	Rapid gelation (<1 min)	Enables fast stabilization during printing	[54]

### 4.1 Disulfide Crosslinking

Disulfide crosslinking occurs through the oxidation of free thiol groups or via thiol–disulfide exchange reactions. In thiolated polymers, crosslinking is achieved by oxidizing attached free thiols, whereas in S‐protected thiolated polymers it proceeds through thiol–disulfide exchange reactions. In the presence of oxygen, thiomers spontaneously form disulfide bonds. Several parameters influence this oxidation process, including the concentration of thiols [43], pH [55], presence of oxidizing agents [44] and the physicochemical properties of the polymeric backbone such as chain length, flexibility, and charge. Because oxygen is already present in hydrogels before the printing process, thiomer‐based hydrogels must be freshly prepared before printing. However, achieving rapid gelation without external triggers during extrusion from single‐channel printing nozzles is challenging. Alternatively, dual‐channel coaxial nozzles enable rapid gelation during printing without additional triggers, as thiomers and highly reactive crosslinkers or oxidizing agents are mixed upon extrusion. Low‐reactivity thiomers, which do not form disulfide bonds before printing, can be activated through the addition of oxidizing agents [56]. The gelation of thiomer hydrogels is accompanied by a decrease in free thiol groups due to disulfide bond formation. This process can be monitored by quantifying the remaining free thiols using Ellman's test [57]. Since the rate of disulfide bond formation depends on the concentration of available thiols, which decreases during crosslinking, the reaction rate gradually slows as gelation progresses [35]. Disulfide bonds can also form through thiol–disulfide exchange reactions, which proceed via a nucleophilic and reversible substitution mechanism independent of oxygen or oxidizing agents [29]. The reaction involves three steps: (i) formation of the thiolate anion, (ii) nucleophilic attack of the sulfur atom on a disulfide via an SN2 mechanism, and (iii) protonation of the resulting thiolate anion [58]. The reaction rate is governed by the pKa of the thiol group, since a lower pKa favors thiolate formation and enhances nucleophilicity [36]. In S‐protected thiomers, thiol–disulfide exchange reactions are triggered by small molecular thiol crosslinkers or endogenous thiols, which induce deprotection of the thiolated moieties on the polymer [12]. The newly liberated thiols subsequently crosslink through thiol–disulfide exchange reactions. For example, S‐protected hyaluronic acid is stable toward oxidation during storage but undergoes thiol–disulfide exchange upon exposure to free thiols such as N‐acetylcysteine or endogenous glycoproteins [45].

### 4.2 Thiol—Ene Crosslinking

**FIGURE 3:**
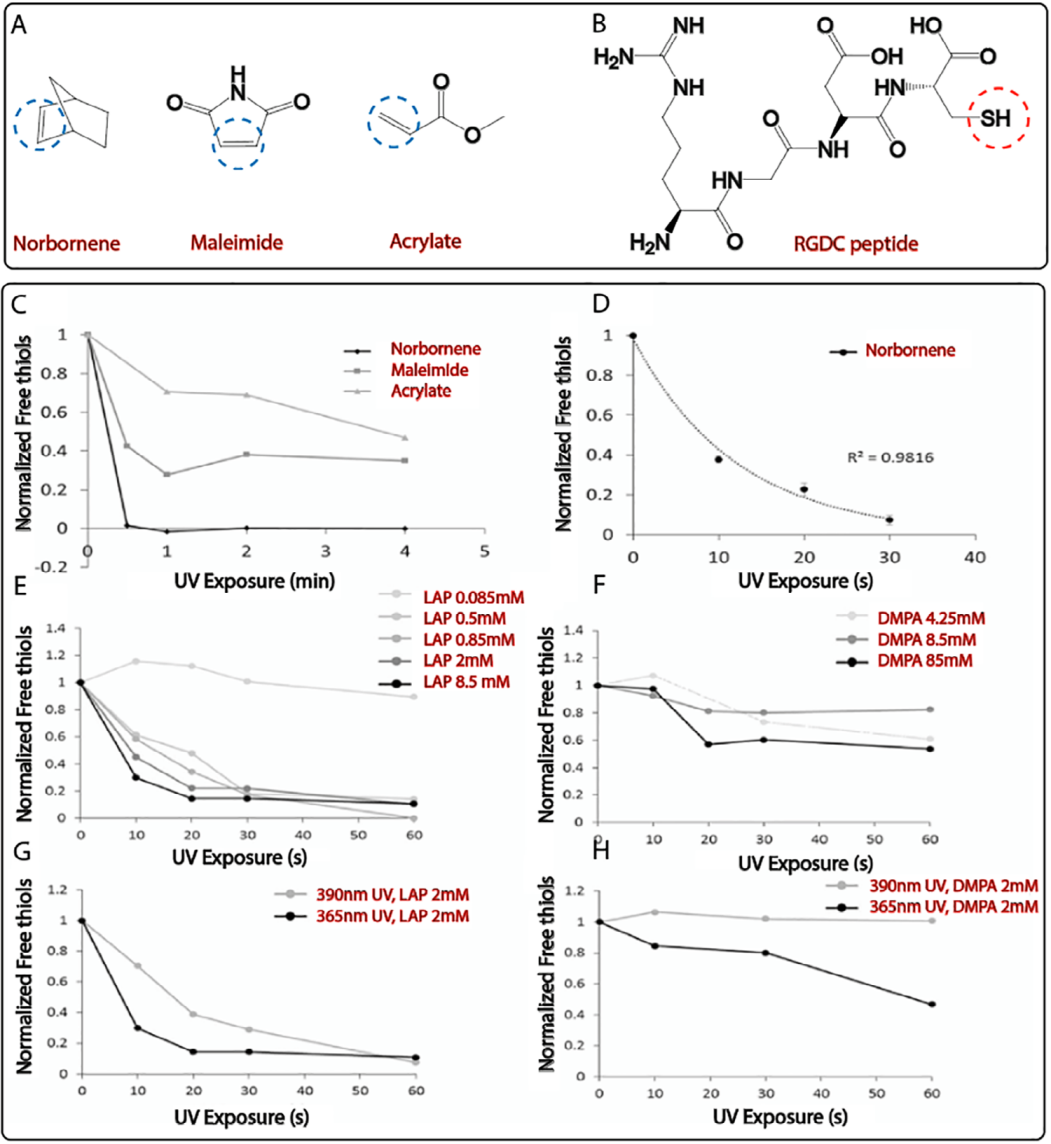
Photochemical reactivity of thiol–ene functional groups and RGDC peptide crosslinking under UV exposure. (a) Chemical structures of representative “ene” functional groups, norbornene, maleimide, and acrylate, used in thiol–ene photopolymerization reactions. (b) Structure of the thiolated peptide RGDC employed for crosslinking reactions. (c) Comparison of normalized free thiols during UV exposure for different “ene” monomers, demonstrating faster consumption in norbornene systems. (d) Kinetic profile and exponential decay fit (*R* ^2^ = 0.9816) of thiol conversion in norbornene‐based reactions. (e–f) Influence of photoinitiator type and concentration, namely 2‐dimethoxy‐2 phenylacetophenone (DMPA) and lithium phenyl‐2,4,6 trimethylbenzoylphosphinate (LAP), on thiol depletion rate under 365 nm UV light. (g, h) Comparison of photoinitiator performance at different UV wavelengths (365 nm vs. 385 nm) indicating wavelength‐dependent efficiency. Data represent mean normalized values (*n* ≥ 3). Adapted and redrawn with permission from [51]. Copyright 2018, Elsevier.

In thiol–ene chemistry, the degree of crosslinking depends on both the nature of the alkene and its concentration. This reaction pathway is particularly attractive because it is not affected by oxygen, and it encompasses two distinct mechanisms: nucleophilic thiol‐mediated Michael additions and radical‐driven thiol–ene processes [59]. The reactivity of the carbon–carbon double bond in these reactions is influenced by the electron‐withdrawing or electron‐enriching effects of neighboring groups. Internal double bonds are generally less reactive than terminal ones. These reactions are typically very rapid, though the reaction rates can be significantly influenced by the reactivity and nature of the selected alkene (“ene” group) [42]. Commonly used “ene” groups include acrylate, methacrylate, allyl, vinyl, norbornene, and maleimide. Michael additions involve nucleophilic additions of thiols to carbon–carbon double bonds [60, 61, 62]. The reaction is catalyzed by compounds, such as bases, that enhance the formation of thiolate anions, which then act as nucleophiles and attack the electrophilic β‐carbon of the double bond. The resulting carbanion withdraws a proton from the conjugate, forming a thioether bond [63]. Poly‐N‐isopropylacrylamide (P‐NiPAAm) copolymers functionalized with thiol and vinyl groups exhibit dual gelation behavior, where physical gelation arises from hydrophobic interactions among the polymer chains and chemical crosslinking occurs through Michael‐type addition between the thiol and vinyl functionalities [46]. Lee et al. and Cheng et al. produced hydrogels by combining P‐NiPAAm co‐polymers modified with acrylates and a four‐armed macromolecule containing four thiol groups [47, 48]. However, the four‐armed crosslinker required high pH to be solubilized and is highly toxic if leached from the material. The strategy from Lee et al. and Cheng et al. was inverted by Robb et al. to eliminate the usage of the toxic crosslinker by synthesizing a thermo‐responsive polymer containing a thiol functionality and instead using a diacrylate PEG polymer to perform the crosslinking [49]. Additionally, thiol‐ene reactions can be triggered by a radical mechanism through the utilization of thermal or photoinitiators that generate radicals or cations under heat or light conditions [50]. In the work by Kim et al., thiolated HA and methacrylated HA were used to prepare an injectable hydrogel through thiol‐ene reaction under initiator and blue light (BL) exposure conditions. The precursor HA reactive solution was injected subcutaneously to porcine skin, which can form a hydrogel by transmitting BL through the skin after injection. Given that UV‐initiated thiol‐ene reactions need a cytotoxic photoinitiator, nucleophilic thiol‐yne reactions are preferred due to their high cytocompatibility. Thiol–norbornene chemistry represents a specific class of thiol–ene reactions in which the ene component is a norbornene moiety. Owing to the high ring strain and unique electronic properties of norbornene, these reactions proceed rapidly and selectively under radical initiation. The mechanism follows the classical radical thiol–ene pathway, where photoinitiators or thermal initiators generate thiyl radicals that add across the norbornene double bond, forming carbon‐centered radicals that propagate through hydrogen abstraction from additional thiol groups. This chain process results in highly efficient thioether crosslinks that are largely insensitive to oxygen inhibition and compatible with mild reaction conditions. Because of these features, thiol–norbornene chemistry has emerged as a powerful tool for polymer network formation, hydrogel fabrication, and bioorthogonal crosslinking in biomedical applications. Dorsey et al. introduced a thiol–norbornene‐based hydrogel platform that enables independent modulation of network stiffness and spatial bioactivity, providing a versatile strategy for designing cell‐responsive, photopatternable matrices. To identify an optimal “ene” functionality for PEG‐based thiol–ene reactions, various PEG monomers bearing distinct unsaturated groups were synthesized, and their reaction kinetics with a thiolated peptide (RGDC) were systematically evaluated (Figure 3) [51].

### 4.3 Thiol—Yne Crosslinking

Thiol–yne chemistry represents an extension of thiol‐mediated click reactions in which thiols react with carbon–carbon triple bonds to form highly crosslinked thioether‐based networks [64]. In contrast to thiol–ene systems, where each unsaturated bond typically reacts with one thiol, thiol–yne reactions can proceed through two sequential thiol additions across a single alkyne [65, 66]. This feature enables the formation of denser polymeric networks, higher crosslinking efficiencies, and enhanced opportunities for tuning the mechanical and structural properties of printed hydrogels. As an example Dolores Ramírez‐Alba et al. developed thiol–yne crosslinked alginate hydrogels for electrically stimulated skin wound healing by crosslinking thiolated alginate with a modified 3‐arm PEG precursor. The resulting hydrogels gelled rapidly within 3 min, exhibited a high gel fraction and structural stability, and, after incorporation of electroactive poly(hydroxymethyl‐3,4‐ethylenedioxythiophene), showed enhanced electrochemical responsiveness together with mechanical properties close to those of skin tissue, supporting their potential as electrode‐like wound dressings for electrically driven skin repair [67]. In a recent pioneering study, Xia et al. developed an injectable alkaline‐responsive dynamic hydrogel for periodontitis therapy via stepwise sulfhydryl click crosslinking between four‐arm PEG‐thiol and alkyne crosslinkers. This click‐mediated network formation enabled efficient rutin encapsulation within the hydrogel matrix, while the presence of labile thioacetate linkages conferred alkaline‐triggered drug release under the pathological gingival crevicular fluid conditions associated with progressing periodontitis [68].

### 4.4 Thiol – Polyphenol Crosslinking

Polyphenols are characterized by at least one benzene ring bearing two or more hydroxyl groups. They can react with thiols to form thioethers through several mechanisms, although Michael‐type additions between polyphenols such as catechol or gallol groups and free thiols are generally preferred [69]. For example, Zhong et al. reported the crosslinking of catechol‐modified hyaluronic acid with 4‐arm‐PEG‐SH, yielding a material that exhibited strong adhesion in aqueous environments as well as self‐healing properties, enabling it to withstand high strain and large deformation [52]. Similarly, Ryu et al. developed a hydrogel by crosslinking catechol‐modified chitosan with a thiolated poloxamer, enabling precise control over gelation speed. The resulting hydrogels displayed excellent gelling behavior and strong tissue adhesion [53]. Compared to disulfide bond formation or most thiol–ene reactions, thiol–polyphenol reactions proceed relatively slowly. However, their rate can be significantly enhanced by the addition of metal ions, oxidizing agents, or enzymes, as discussed in detail in Section 6. For instance, chitosan derivatives containing both thiol and catechol groups did not undergo crosslinking under mild conditions, but upon addition of sodium periodate, rapid gelation (<1 min) was achieved [54].

## 5 Printability Metrics and Rheological Performance

Thiolated polymers can be 3D printed by extrusion, inkjet, and vat‐polymerization approaches. Extrusion bioprinting deposits continuous filaments of cell‐laden bioinks layer‐by‐layer to form constructs. From an equipment perspective, extrusion‐based printing of thiolated bioinks is typically performed using pneumatic or piston‐driven bioprinters [70, 71, 72, 73, 74, 75, 76, 77, 78], where printing fidelity depends on the interplay between shear‐thinning behavior, nozzle diameter (typically 100–400 µm), and gelation kinetics [79, 80, 81]. In these systems, thiolated polymers are particularly advantageous due to their ability to undergo in situ crosslinking via disulfide formation or thiol–disulfide exchange, allowing stabilization of printed filaments post‐deposition. However, excessive premature gelation can compromise printability, necessitating careful control of oxidation conditions and formulation timing. Key parameters include extrusion pressure, printing speed, and gelation rate, which must be balanced to maintain filament continuity and shape retention. While versatile and compatible with high‐viscosity thiolated polymers like thiolated polysaccharides, its relatively low resolution (>100 µm) and slower fabrication limit the recreation of fine tissue microarchitecture. By using coaxial nozzles, a crosslinking or oxidizing solution can be delivered simultaneously with the thiolated polymer, markedly improving printing resolution [82].

Inkjet bioprinting, in contrast, dispenses discrete droplets of low‐viscosity bioinks using thermal or piezoelectric actuation. This enables higher throughput and finer placement resolution, making it well‐suited for patterning cells, growth factors, or gradients. Generally, this technique is suitable for thiomers of comparatively low molecular mass or highly diluted thiomers, as only low‐viscosity bioinks can be used. Further limitations are a potential cell stress during droplet formation and challenges in building large, mechanically robust 3D structures.

Vat‐polymerization methods, including stereolithography (SL) and digital light processing (DLP), use patterned light to photopolymerize liquid bioinks into 3D constructs with high resolution and speed. These methods rely on spatially controlled light‐induced curing of photosensitive resins and enable the fabrication of complex architectures with significantly higher resolution and throughput compared to nozzle‐based systems [83, 84, 85]. Vat photopolymerization systems enable layer‐by‐layer curing of entire cross‐sections, significantly enhancing printing throughput [86, 87, 88]. These systems can achieve lateral resolutions of approximately 20–50 µm, with advanced systems reaching below 20 µm, and layer thicknesses in the range of 10–100 µm depending on resin reactivity and light penetration depth. The rapid kinetics of thiol–ene reactions reduce exposure time per layer, enabling faster build speeds while maintaining high structural fidelity. Importantly, the step‐growth mechanism of thiol‐based photopolymerization delays gelation and reduces internal stress accumulation, minimizing defects such as warping, cracking, and inhomogeneous curing, which are commonly observed in chain‐growth acrylate systems. The decoupling of rheological constraints from printability in DLP systems represents a significant advantage for thiolated polymers, enabling the use of low‐viscosity formulations while maintaining precise spatial control over crosslinking. Several studies have demonstrated the suitability of thiol‐based systems for DLP printing. For example, Fairbanks et al. reported highly uniform thiol–ene photopolymer networks with rapid curing kinetics and reduced oxygen inhibition, enabling precise feature fabrication under mild irradiation conditions [89]. Similarly, Arickx et al. demonstrated thiol–ene hydrogels with tunable mechanical properties and cytocompatibility, highlighting their applicability for biofabrication [90]. More recently, biobased thiol–ene resins derived from polyesters and multifunctional thiols have been successfully processed using DLP to fabricate complex geometries, including porous scaffolds and mechanically responsive architectures, confirming the compatibility of thiolated systems with high‐resolution vat photopolymerization platforms [91].

In Table 4 an overview about the different 3D bioprinting techniques that can be used for thiolated polymers is provided.

**TABLE 4:** Comparison of different 3D bioprinting techniques that can be used for thiolated polymers [92, 93, 94].

	Extrusion bioprinting	Inkjet bioprinting	Vat‐polymerization bioprinting
**Printing mode**	Layer‐by‐layer	Drop‐by‐drop	Layer‐by‐layer
**Processing speed**	Slow (10–50 µm/s)	Fast (1–10 000 droplet s^−1^)	Fast (<1 h)
**Viscosities of bioinks**	30 mPa s^−6^ × 10^7^ mPa s	<10 mPa s	No limitation
**Cell viability**	80%–90%	80–95%	>90%
**Cell density**	High density (>10^8^ cells/ mL)	Low cell density (<16 × 10^6^ cells/mL)	Medium cell density (<10^8^ cells/mL)
**Resolution**	>100 µm	50 µm	20–100 µm
**Quality of printed construct**	Good	Poor	Good
**Printer cost**	Moderate	Low	Low

**FIGURE 4:**
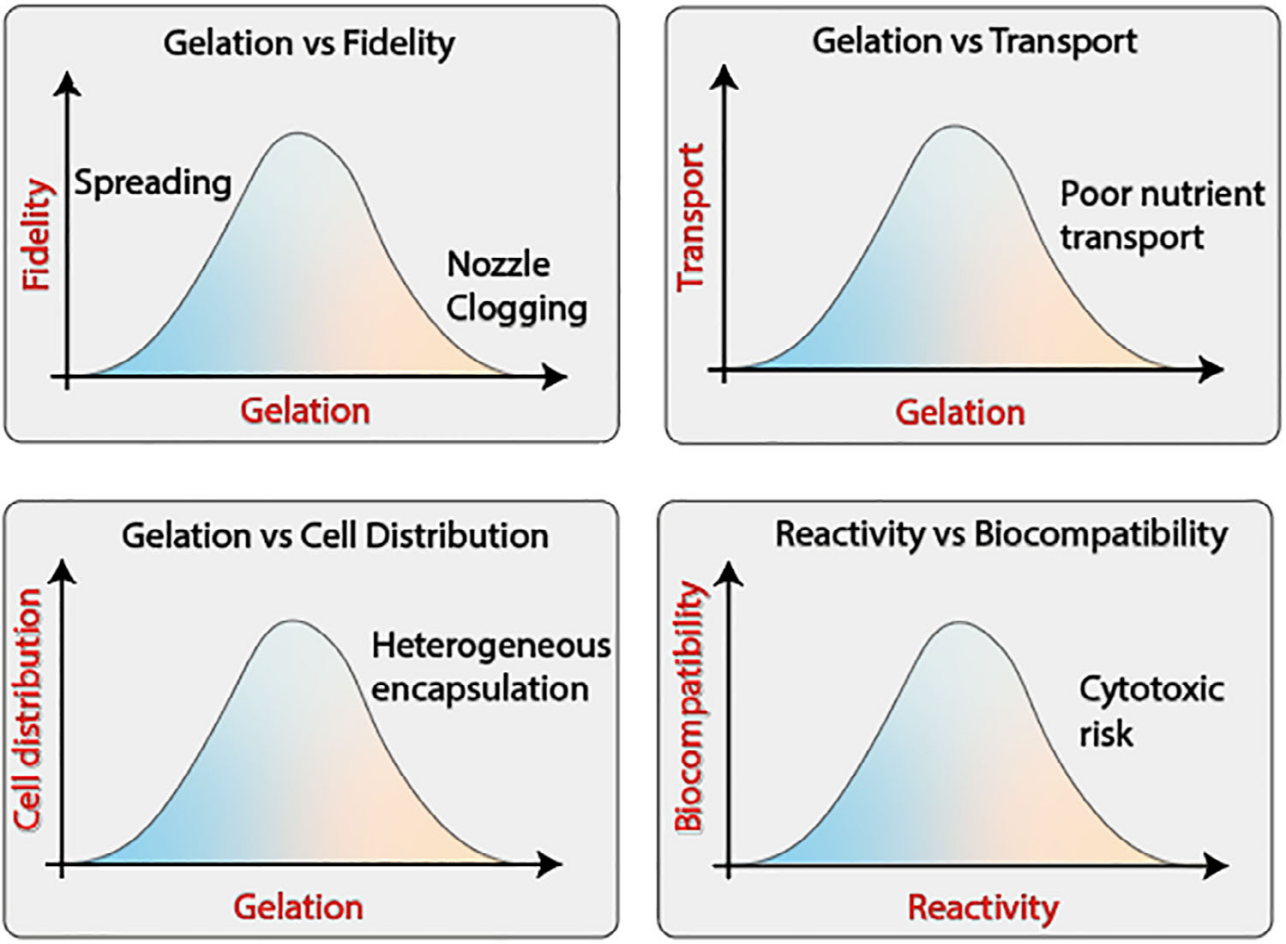
Trade‐off landscape governing thiolated bioink performance in 3D bioprinting.

The translation of thiolated polymers into functional bioinks depends on their rheological and gelation behaviour, which govern extrusion dynamics, filament fidelity, and cell viability. Quantitative assessment of these parameters is essential for rationally designing thiomer‐based inks optimized for 3D bioprinting. Extrusion‐based printing requires materials that behave as non‐Newtonian, shear‐thinning fluids [95, 96]. The apparent viscosity (η) should decrease with increasing shear rate (γ̇), typically following the power‐law relation *η* = Kγ̇^(n−1), where *n* is the flow index [97, 98]. Printable thiomer hydrogels generally exhibit pronounced shear‐thinning behavior, which facilitates smooth extrusion while minimizing pressure build‐up and shear‐induced cell damage. The viscosity at low shear rates (10–100 s^−^^1^) governs shape retention, whereas values at high shear rates (500–1000 s^−^^1^) reflect printability through narrow nozzles [99]. Optimal pre‐print viscosities generally lie between 30–300 Pa·s, balancing extrudability and structural fidelity [100]. A minimum yield stress (τ_y) of 20–80 Pa is required to prevent sagging or spreading after deposition [6]. Below this threshold, filaments collapse under their own weight, while excessively high τ_y values (>150 Pa) can cause discontinuous flow or nozzle clogging [24]. Dynamic oscillatory rheology provides additional insights through the storage (G′) and loss (G″) moduli, which describe elastic and viscous contributions, respectively [101]. For printable thiomer inks, the ratio G′/G″ (tan δ) should fall between 0.3 and 1 before printing, enabling the transition from viscous to elastic behavior during or immediately after extrusion [102]. Quantitative metrics such as strand collapse angle, filament fusion index, and fidelity score provide standardized means to assess structural accuracy [81, 103]. Assessment of thiomer‐based bioinks requires consideration of both printability and biocompatibility. To summarize the multidimensional design constraints of thiomer‐based bioinks, Figure 4 presents a design‐space framework illustrating how gelation must be carefully balanced against printing fidelity, nutrient transport, and cell distribution, while reactivity must be optimized to preserve biocompatibility.

Key parameters such as collapse angle, filament fusion index, and fidelity ratio define shape retention and structural accuracy, while crosslinking kinetics, through disulfide exchange, thiol–ene, or Michael addition, control gelation time, modulus buildup, and mechanical integrity. Ideal formulations exhibit rapid gelation (30 s–3 min) and moderate shear rates that preserve cell viability above 80%–90%. Further information is summarized in Table 5, which outlines the key performance indicators and optimal ranges for evaluating thiomer‐based bioinks.

**TABLE 5:** Key parameters governing printability, crosslinking, and biocompatibility of thiolated polymer‐based bioinks.

Parameter	Definition	Optimal	Key influence
Collapse angle test	Measures filament deflection under gravity.	< 30°	Indicates filament stability; lower angles denote stronger structural integrity.
Filament fusion index	Quantifies strand coalescence (ratio of fused to theoretical distance).	0.8–1.2	<0.8: weak bonding; >1.2: over‐gelation and loss of resolution.
Fidelity Ratio (F = A_p_/A_t_)	Compares printed vs. theoretical pore areas (via optical/confocal imaging).	0.8–1.0	Reflects shape accuracy and print fidelity.
Crosslinking kinetics	Governed by disulfide, thiol–ene, or Michael addition reactions.	—	Determines print resolution, gel strength, and mechanical stability.
Gel Point (t_gel)	Time at which the storage modulus (G′) equals loss modulus (G″) during rheology.	30 s–3 min	Ideal for bioprinting; ensures timely structural stabilization.
Modulus Build‐Up Rate (dG′/dt)	Rate of network formation (Pa·s^−^^1^).	> 10 Pa·s−^1^	Higher rates improve filament stacking and layer adhesion.
Oxygen inhibition	Suppression of radical‐based thiol–ene reactions by O_2_.	Minimized under humidified/inert conditions	Prevents incomplete curing and ensures consistent gelation.
Extrusion pressure/Shear rate	Physical stress during printing.	< 1000 s^−^^1^	Excessive shear induces ROS generation and cell apoptosis.
Cell Viability (24 h post‐print)	Percentage of live cells after printing.	> 80%–90%	Ensures biocompatibility and minimal oxidative stress.

## 6 Strategies to Trigger and Accelerate Crosslinking in 3D Bioprinting

The development of robust, high‐resolution 3D bioprinted constructs relies heavily on controlling the speed and efficiency of hydrogel crosslinking. For thiolated polymers, gelation must be rapid enough to stabilize deposited filaments yet sufficiently controllable to prevent premature clogging or nozzle blockage. A variety of strategies have been established to trigger and accelerate crosslinking during or immediately after the printing process. These include the addition of low‐ and high‐molecular‐weight crosslinkers, light‐activated thiol–ene chemistry, oxidizing agents, enzymatic reactions, and dual‐stage hybrid systems.

### 6.1 Addition of Crosslinkers

The crosslinking of thiomers can be triggered by the addition of small or polymeric crosslinkers via coaxial nozzles during the printing process. Table 6 provides a direct comparison of crosslinker classes, highlighting their respective strengths and limitations. Critical consideration is thereby the potential for heterogeneity; rapid gelation by small molecules ensures uniform but softer gels, whereas the slower diffusion of polymeric crosslinkers may generate stiffness gradients, which can be detrimental to reproducibility yet advantageous for mimicking natural tissue zonation. Importantly, hybrid strategies that combine both small and polymeric crosslinkers, such as initiating gelation with small‐molecule thiols for immediate stabilization, followed by reinforcement with multi‐arm PEGs, offer a route to finely balance printability, mechanical robustness, and biological function in thiolated bioinks.

**TABLE 6:** Comparison of crosslinker types employed in thiolated polymer‐based bioinks.

Crosslinker	Examples	Gelation kinetics	Properties	Biological effects	Advantages	considerations
Small‐molecule thiols	Dithiothreitol (DTT)	Very rapid (seconds–minutes); diffusion‐controlled; suitable for coaxial or in situ activation	Form uniform but softer, less elastic networks	Can modulate intracellular redox state; influence cell proliferation and differentiation via GSH/ROS balance	Fast reaction; controllable redox responsiveness; compatible with physiological conditions	Over‐gelation or premature crosslinking; limited long‐term mechanical stability; potential ROS generation
Polymeric/multi‐arm PEG crosslinkers	4‐arm PEG‐SH, 4‐arm PEG‐maleimide, PEG‐acrylate	Moderate (minutes–tens of minutes); reaction limited by chain mobility	Produce stronger, more elastic and durable networks	Bioinert by default; tunable by conjugation of RGD or growth factors	High mechanical robustness; excellent tunability; good cytocompatibility	Slower diffusion may yield stiffness gradients; requires functionalization chemistry
Hybrid/sequential systems	Small‐molecule + PEG (dual‐stage), PEG‐GSH, PEG‐NAC systems	Two‐step kinetics: fast initial stabilization + slow reinforcement	Balanced elasticity and viscoelastic recovery; improved long‐term integrity	Enables spatial and temporal control of cell–matrix interactions	Combines advantages of both types; supports print fidelity and remodelling	More complex formulation; difficult to optimize stoichiometry and timing

### 6.2 UV‐Light and Activators

Frequently used photochemical initiators for thiol‐ene reactions are acetophenone compounds such as 2,2‐dime‐thoxy‐2‐phenyl acetophenone, lithium phenyl‐2,4,6‐trimethyl benzoyl phosphinate, or Eosyn‐Y, forming radicals under visible light [104]. After initiation, the reaction proceeds through the attack of the thiyl radical on the alkene to form a new carbon radical [105]. This carbon radical reacts with another thiol substrate so that a thioether and a new thiyl radical are formed. The latter ones allow the propagation step to continue the cycle. The most often used photo‐chemically initiated reaction is that one with norbornene as alkene moiety. It is a stable strained cyclohexane ring with a methylene bridge that reacts with thiol groups [106]. Norbornene exhibits a very high reactivity toward thiol‐ene reactions [107]. Allyl, vinyl, and NOR groups, exhibit higher selectivity in thiol‐ene reactions without significant interference from radical pathways [108]. This selectivity ensures cleaner reactions and higher yields of thiol‐ene adducts. Particularly, it has been demonstrated that, when exposed to light, the NOR groups react exceptionally fast (within seconds) with ─SH groups through a step‐growth polymerization mechanism [109] to the high reactivity and specificity between the correspondent radicals, as well as the high thermodynamic driving force.

### 6.3 Oxidizing Agents

The introduction of oxidizing agents during the printing process has emerged as one of the most effective ways to accelerate disulfide crosslinking in thiolated polymers [110]. This strategy is particularly compatible with coaxial bioprinting, in which the thiolated bioink is extruded from the core nozzle while the oxidizing solution flows through the sheath [111, 112]. By separating the reactive components until the moment of extrusion, gelation can be triggered in a controlled manner precisely at the point of deposition, thereby preventing premature crosslinking within the nozzle and ensuring rapid filament stabilization once the construct is printed. The underlying mechanism relies on the ability of oxidants to convert free thiols into disulfide bonds. Depending on the strength and concentration of the oxidant, this process can proceed within seconds to minutes and is accompanied by a marked increase in viscosity. Classic examples include hydrogen peroxide, sodium periodate, ammonium persulfate, sodium hypochlorite, and carbamate peroxides, which act as efficient small‐molecule oxidants capable of diffusing through the polymer network and inducing rapid covalent stabilization. In one representative case, the addition of sodium periodate to thiolated chitosan led to more than a 10 000‐fold increase in viscosity within minutes, demonstrating the remarkable capacity of oxidants to accelerate gelation [44]. The advantages of oxidant‐mediated gelation lie in its speed, simplicity, and broad applicability across different thiolated polymer backbones, ranging from natural polysaccharides such as chitosan and HA to synthetic acrylates. At the same time, this approach faces several limitations that must be carefully managed. Strong oxidants may generate reactive oxygen species, which, in excess, can impair cell viability and compromise long‐term functionality [113, 114]. Over‐crosslinking can also produce brittle gels with poor remodelling capacity, while the bulk nature of oxidation reactions offers less spatial precision compared to light‐activated approaches [113, 115]. Despite these challenges, oxidizing agents have established themselves as a practical and versatile means of controlling gelation in 3D bioprinting. By adjusting the type and concentration of oxidant, it is possible to fine‐tune gelation kinetics and mechanical properties to suit different printing modalities. When combined with coaxial extrusion, where the oxidant is introduced only upon deposition, this method achieves a balance between rapid structural stabilization and preservation of cell viability, making it an increasingly important tool in the formulation of thiolated bioinks.

### 6.4 Enzymes and Redox‐Mediators

**FIGURE 5:**
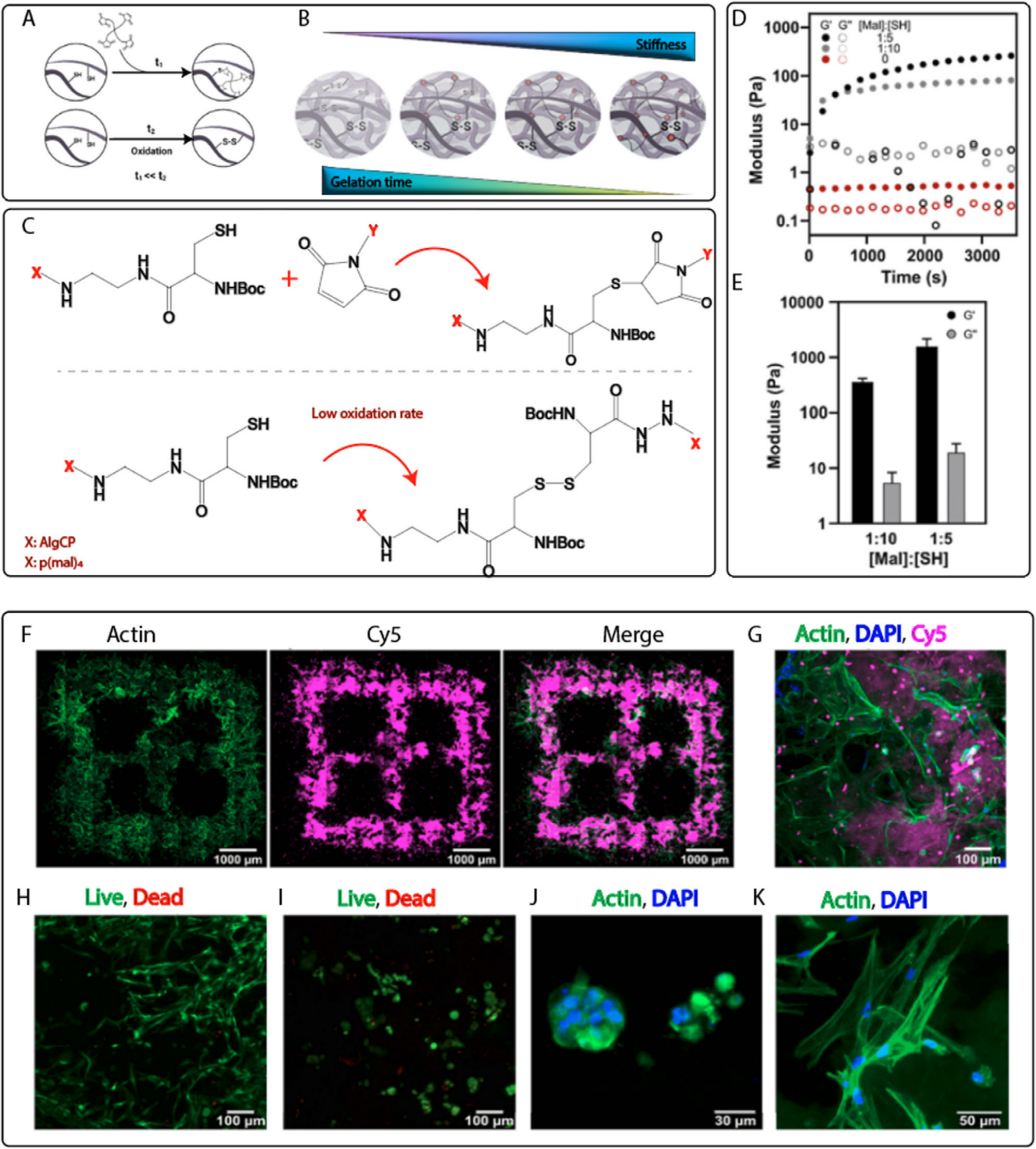
(A and B) Schematic of GSH‐responsive hydrogels cross‐linked via disulfides or combined disulfide–p(Mal)_4_ networks.(C) Schematic representation of AlgBCP (+PGA) cross‐linking using p(Mal)_4_ or intermolecular disulfides. (D) Cross‐linking kinetics of 1% (w/v) AlgBCP (+PGA) by either pMal_4_ or disulfide formation. (E) Shear modulus of AlgBCP (+PGA) hydrogels cross‐linked using p(Mal)_4_. (F and G) Confocal micrographs of bioprinted fibroblast cells embedded in AlgCP (+PGA) cross‐linked using p(Mal)_4_ ([Mal]:[SH] ratio of 1:10) seven days post printing. Live‐dead staining revealed (H) 98% cell viability for fibroblast cells and (I) 90% for MCF7 cells post‐printing. (J) MCF7 cells form spheroids in the printed structures, whereas (K) fibroblasts showed an extended morphology. Adapted and redrawn with permission from [123]. Copyright 2023, Elsevier.

Enzymatic and redox‐mediated strategies have emerged as attractive approaches to accelerate crosslinking in thiolated bioinks because they operate under mild, physiologically compatible conditions, avoiding the need for harsh oxidants or high‐energy light exposure that may compromise cell viability [116, 117]. These systems take advantage of naturally occurring catalytic pathways or redox‐active molecules to drive the formation of disulfide bonds or thiol‐mediated couplings, thereby enabling dynamic and cytocompatible gelation suitable for cell‐laden constructs [118]. A widely studied example is the horseradish peroxidase (HRP)/hydrogen peroxide (H_2_O_2_) system, which catalyzes thiol oxidation at physiological pH [118]. In this mechanism, HRP uses H_2_O_2_ as a co‐substrate to generate reactive radical intermediates that rapidly oxidize thiol groups into disulfides, resulting in stable crosslinked networks [119, 120]. This approach offers tunable kinetics: varying the concentration of H_2_O_2_ or HRP can accelerate or slow down the gelation rate, providing precise control over hydrogel formation. Importantly, when optimized, the concentrations of H_2_O_2_ required are sufficiently low to maintain cell viability, making HRP‐mediated crosslinking highly attractive for in situ bioprinting and injectable hydrogel applications [121, 122]. In addition to peroxidases, oxidoreductases like laccase and tyrosinase oxidize catechol or gallol groups to highly reactive o‐quinones, which readily undergo nucleophilic attack by thiols to form covalent thioether bonds. This reaction mechanism has been exploited in the design of mussel‐inspired adhesive hydrogels, where catechol‐modified polymers are crosslinked with thiolated counterparts in the presence of laccase or tyrosinase [121]. The resulting hydrogels display strong tissue adhesion and rapid gelation in aqueous environments, along with tunable mechanical properties. These systems are particularly relevant for 3D bioprinting of tissue adhesives or bioinks designed for integration with soft, wet biological substrates. Naeimipour et al. introduced a bioorthogonal, enzyme‐controlled thiol chemistry to achieve dynamic covalent cross‐linking in carbohydrate‐based hydrogels, addressing the issue of uncontrolled thiol oxidation. In their design, alginate was functionalized with cysteine groups shielded by an enzyme‐cleavable thiol‐protecting moiety (Phacm). Upon treatment with penicillin G acylase, the Phacm group was removed, liberating free thiols. These thiols then underwent oxidation under physiological conditions, leading to reversible cross‐link formation and generation of hydrogels with adjustable mechanical stiffness [116]. In a related work, Naeimipour et al. constructed an alginate‐based hydrogel (Alg) functionalized with phenylacetamidomethyl (Phacm)–protected cysteine residues, allowing controlled thiol activation upon enzymatic cleavage. While the resulting disulfide‐mediated cross‐linking proceeds more slowly than the rapid gelation achieved with 4‐arm‐PEG‐maleimide (pMal_4_), these disulfide bonds continue to accumulate over time and gradually increase the stiffness of the hydrogel. Moreover, retaining the Boc protecting group on the cysteine N‐terminus further suppresses thiol reactivity, reducing the likelihood of premature oxidation and disulfide formation. Cross‐linking of alginate functionalized with Boc‐protected Cys‐Phacm (AlgBCP) using pMal_4_, following PGA‐mediated removal of the Phacm group, produced a storage modulus comparable to that of AlgCP. The selective deprotection by penicillin G acylase (PGA) generated free thiols on demand, allowing controlled hydrogel crosslinking through thiol‐reactive linkers and intermolecular disulfide formation while minimizing unwanted thiol oxidation. The remaining thiol groups further enabled post‐printing functionalization and layer‐by‐layer lamination (Figure 5). Additionally, incorporating PGA into a thermo‐reversible support bath facilitated the bioprinting of cell‐laden 3D constructs with high viability and excellent structural precision [123].

Beyond enzymatic catalysis, redox mediators provide another route to accelerate or regulate thiol crosslinking. Molecules such as ferrous ions (Fe^2^^+^), glutathione (GSH), and other small redox‐active species can directly modulate the thiol/disulfide equilibrium [124]. The thiol–disulfide exchange plays a fundamental role in numerous biological mechanisms, including the proper folding of proteins. Hydrogels stabilized through disulfide crosslinks exhibit rapid gelation behavior, making them suitable for applications like cell encapsulation [125]. Such reversible systems allow printed constructs to remodel in response to cellular activity and environmental changes, mimicking the dynamic behavior of the native extracellular matrix [126, 127]. Various cell types, including immortalized hepatocytes, vascular endothelial cells, and mesenchymal stem cells, have demonstrated the ability to remain viable and proliferate within disulfide‐crosslinked hydrogels. Moreover, these cells can be efficiently recovered by dissolving the hydrogels through thiol–disulfide exchange reactions [128, 129]. Similarly, metal ion–mediated redox reactions can facilitate localized and rapid thiol oxidation, although care must be taken to prevent cytotoxic effects associated with high concentrations of transition metals [130]. Overall, enzymatic and redox‐mediated crosslinking strategies offer a unique balance between biocompatibility, controllability, and functionality. Unlike photoinitiated approaches, they do not require external light or photoinitiators, reducing the risk of cytotoxicity. Moreover, the reversibility of redox‐mediated systems introduces self‐healing and remodelling capabilities that are difficult to achieve with purely covalent networks. However, these methods often exhibit slower reaction kinetics compared to radical‐mediated thiol–ene systems, which may limit their use in high‐throughput or rapid bioprinting settings. Ongoing work is focused on engineering enzyme formulations and redox mediators that combine rapid gelation with the mild conditions required for sensitive cell types.

### 6.5 Dual‐Stage and Hybrid Crosslinking Approaches

Single crosslinking mechanisms often fail to meet all the requirements of 3D bioprinting, as they rarely provide both the rapid initial stabilization needed to maintain filament shape and the long‐term mechanical robustness required for functional tissue constructs. To overcome this limitation, dual‐stage and hybrid crosslinking approaches have been developed. These strategies combine orthogonal chemistries or sequential mechanisms, enabling bioinks to gel quickly during deposition while continuing to strengthen, remodel, or stabilize over time [131]. Such modular systems allow researchers to precisely tailor gelation kinetics and mechanical properties to match the demands of different tissue engineering applications. One illustrative approach involves alginate–thiomer bioinks, which exploit the rapid ionic gelation of alginate with divalent cations such as calcium. This ionic crosslinking provides immediate filament stabilization during printing, preventing collapse of the construct and ensuring shape fidelity. However, ionically crosslinked alginate gels alone are often mechanically weak and prone to degradation. To address this, the system incorporates thiolated polymers that subsequently undergo covalent crosslinking via disulfide formation or thiol–ene chemistry, providing a secondary network with enhanced durability and long‐term stability. The combination of fast ionic and slower covalent crosslinking yields scaffolds that are both printable and structurally resilient, making them suitable for load‐bearing tissues. Another example of hybrid crosslinking leverages the orthogonality of photocrosslinking and disulfide chemistry. In this system, thiol–norbornene groups undergo rapid radical‐mediated photopolymerization immediately after deposition, producing a highly crosslinked network that stabilizes the construct within seconds. Over time, the same thiolated groups continue to participate in thiol–disulfide exchange reactions, introducing reversible bonds that allow for gradual remodelling and self‐healing [7]. This dual mechanism ensures that constructs not only maintain their integrity during and after printing but also retain the dynamic adaptability necessary for long‐term tissue integration and regeneration. A further example arises from polyphenol–thiol systems. Initially, catechol groups engage in non‐covalent interactions such as hydrogen bonding and π–π stacking with surrounding polymers, imparting immediate shape fidelity and adhesive properties to wet surfaces [53]. These reversible interactions are later reinforced by oxidative coupling of catechols and thiols, producing robust covalent thioether linkages that lock the network into place. This dual‐stage process provides hydrogels that are not only printable and adhesive but also mechanically stable under physiological conditions, with potential applications in wound sealing and tissue adhesion. The major advantage of such dual‐stage and hybrid systems lies in their modular control over gelation times and mechanical evolution. Rapid crosslinking mechanisms, ionic interactions, photopolymerization, or non‐covalent binding, provide the immediate stability required for extrusion fidelity. Slower processes, disulfide exchange, oxidative coupling, or secondary covalent crosslinking, reinforce the network, enhance mechanical robustness, and introduce remodelling capabilities. This combination ensures that thiolated bioinks can achieve both printability and post‐print functionality, addressing one of the central challenges in the bioprinting of complex, cell‐laden constructs. By providing both strong interfacial bonding and responsiveness to redox conditions, thiomers offered a unique balance between stability and adaptability, which soon shifted their role from being regarded solely as excipients toward broader recognition as functional biomaterials [132, 133]. This recognition accelerated their exploration as ECM‐mimetic hydrogels. Beyond adhesion, the ability of thiolated polymers to crosslink in situ generated hydrated, viscoelastic networks with tunable stiffness and degradation rates, properties essential for cell–matrix interactions [134, 135]. Importantly, their intrinsic capability for dynamic disulfide exchange distinguished them from many synthetic hydrogels by introducing reversible remodelling and self‐healing features. The emergence of 3D bioprinting provided a new arena in which the chemical versatility of thiomers could be leveraged [136, 137]. The demand for bioinks with rapid yet controllable gelation kinetics, high shape fidelity, and cytocompatibility created an ideal application space [6, 99, 138]. Additionally, glutathione‐driven redox remodelling supports self‐healing and long‐term functionality. As glutathione is capable of participating in thiol–disulfide exchange reactions, it drives not only crosslinking but also introduces a reversible and dynamic character to the hydrogel network [127, 139, 140]. Beyond their influence on gelation speed and mechanical integrity, crosslinkers also shape the biological microenvironment within printed constructs [81]. For instance, small‐molecule thiols such as glutathione not only accelerate network formation but can also participate in cellular redox regulation, thereby influencing signaling pathways relevant to proliferation and differentiation [141]. In contrast, multi‐arm PEG crosslinkers are largely bioinert but offer the advantage of being easily functionalized with bioactive peptides (e.g., RGD), enabling incorporation of cell‐adhesive sites within otherwise inert matrices [105, 142]. As constructs are printed layer‐by‐layer dual‐stage crosslinking is also essential to guarantee a proper connection between each layer. Fast initial crosslinking guarantees sufficient gelation whereas the following slow crosslinking provides the binding between each layer. In addition, the self‐healing of thiomers via thiol/disulfide exchange reactions contributes to this binding.

## 7 Biomedical Applications of 3D Bioprinted Thiomers

**FIGURE 6:**
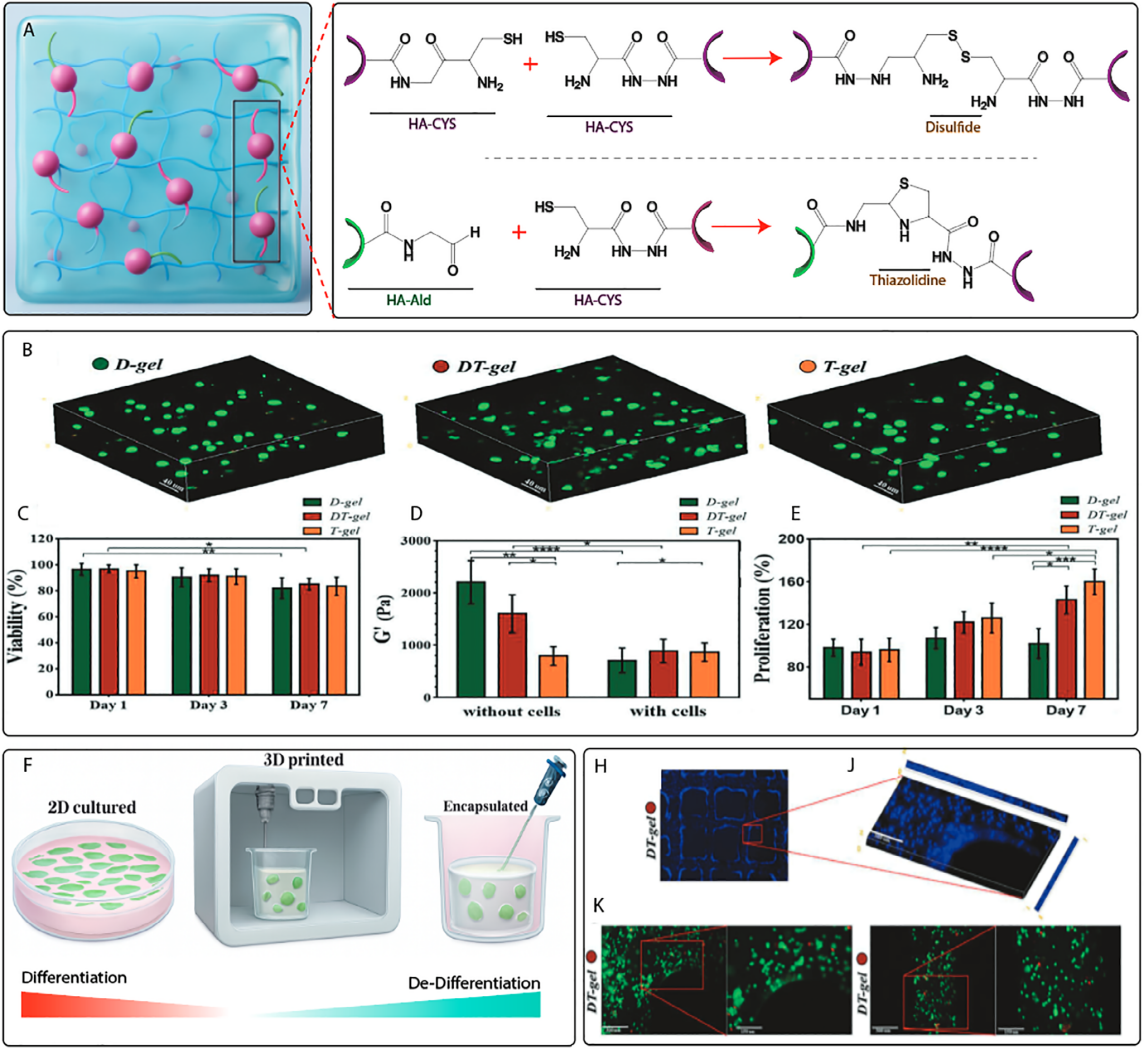
(A) Illustration of HA‐Cys and HA‐Ald reacting to form disulfide and thiazolidine linkages at varying ratios. (B) 3D reconstruction of encapsulated hMSCs in hydrogels after 1 day, cells are stained with green (live) and red (dead) stains to determine cell viability; (C) Viability of hMSCs in hydrogels over time calculated from microscopic images of Live/Dead stained hydrogels; (D) Storage modulus of hydrogels after 3 days incubation in cell culture media with and without encapsulated cells; (E) Proliferation of encapsulated cells over time determined by PrestoBlue assay. (F) Schematic representation of the impact of 2D and 3D on the stemness of hMSCs. (H and J) Microscopic image of printed Hoechst‐stained hMSCs using the hydrogel inDT‐gel group; (K) Microscopic images of hMSCs printed through DT‐gel and stained by Live/Dead kit, presenting two different areas of structure with two magnifications. Adapted and redrawn with permission from [146]. Copyright 2024, Wiley‐VCH GmbH.

Thiomers constitute a broad class of redox‐active biomaterials that have transformed the design of injectable, printable, and self‐healing hydrogels. Their reversible disulfide, thiol‐ene, and thiol‐Michael reactions enable in situ gelation, mechanical tunability, and dynamic remodelling under physiological conditions. These properties make thiomer inks particularly attractive for soft‐tissue engineering, ocular therapy, wound healing, and stem‐cell scaffolding. Their chemistries are compatible with diverse polymer backbones, including hyaluronic acid, chitosan, dextran, PEG, gelatin, and collagen, allowing customizable cross‐linking and biological functionality. Hydrogels derived from thiolated hyaluronic acid (HA) have been widely applied for ophthalmic and articular repair [6, 143]. Disulfide‐cross‐linked or thiol‐ene–modified HA networks allow controlled gelation essential for ocular administration, where rapid cross‐linking prevents drug loss from the eye surface [144, 145]. Tavakoli et al. developed an innovative extracellular matrix–based bioink for human mesenchymal stem cells (hMSCs) by modifying HA with cysteine and aldehyde groups, enabling dual crosslinking via disulfide and thiazolidine bonds (Figure 6). This dual‐network design enhanced hydrogel stability and biological performance, yielding rapid gelation, shear‐thinning behavior, and strong shape retention. The bioink maintained high cell viability post‐printing, promoted proliferation and migration, and increased stemness marker expression (OCT3/4 and NANOG) by over twofold. Disulfide bonds facilitated self‐healing and cell motility, while thiazolidine linkages accelerated gelation and improved long‐term structural integrity [146].

**FIGURE 7:**
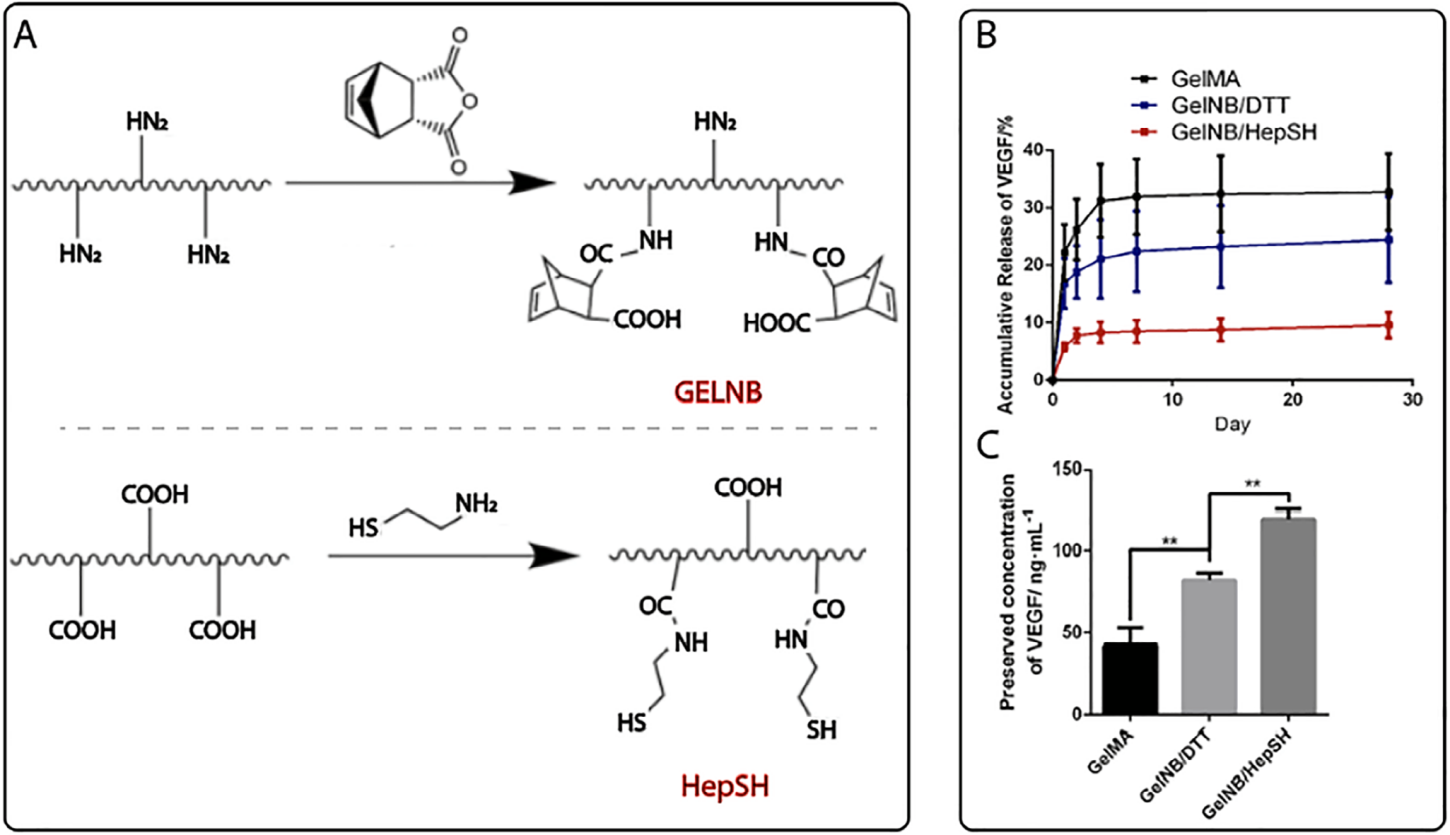
(A) Synthesis of GelNB and HepSH. (B) Controlled release of VEGF from GelMA, GelNB/DTT, and GelNB/HepSH hydrogels, tested by ELISA assay. (C) Concentration of preserved VEGF measured after 28 days of release and hydrogels hydrolyzed by type II collagenase. Adapted and redrawn with permission from [154]. Copyright 2021, American Chemical Society.

Hybrid systems combining thiolated chitosan (CS‐NAC) with silk fibroin have been designed for cartilage tissue engineering [147]. The first network (tyrosine‐cross‐linked silk fibroin) confers elasticity, while disulfide bonds within the CS‐NAC network add stiffness and resilience. Other double‐network hydrogels linking oxidized dextran and thiolated chitosan through Schiff‐base and disulfide bonds offered high mechanical integrity and biocompatibility [148]. The dynamic redox responsiveness of thiomer inks facilitates controlled porosity and gradient stiffness, critical for the formation of vascularized tissues. HA‐PEG disulfide hydrogels and thiol‐ene PEG–gelatin scaffolds can be engineered to promote endothelial invasion and lumen formation. Incorporating angiogenic factors such as VEGF or FGF‐2 within these matrices enhances vascular sprouting, while thiolated HA decorated with cell‐adhesive peptides (e.g., RGD) fosters endothelial alignment and integration with stromal cells. Thiolated PEG has also been used as a cross‐linker with maleimide‐functionalized PEG, producing injectable hydrogels for sustained ocular delivery of Avastin (bevacizumab), achieving therapeutic release for up to 14 days in models of intraocular neovascularization [149]. Multifunctional thiolated PEGs further serve as cross‐linkers for vinyl‐sulfone‐functionalized dextran, forming hydrogels capable of sustained protein release (immunoglobulin, lysozyme, bFGF) over controlled timeframes [150]. Thiomers exhibit intrinsic tissue adhesion through disulfide bond formation with cysteine‐rich proteins at wound interfaces, ensuring rapid hemostasis and conformal coverage. Thermo‐responsive thiolated chitosan/β‐glycerophosphate (β‐GP) systems form injectable hydrogels that gel at body temperature via physical and chemical crosslinking, yielding high mechanical strength and prolonged retention [151]. When loaded with the Histatin‐1 peptide, these hydrogels enhanced epithelial migration and angiogenesis, achieving 84% wound closure within 7 days [152]. Thiolated chitosan–hydroxyapatite–β‐GP composites allow sustained release of BMP‐2 for bone repair, while PEG‐grafted thiolated chitosan crosslinked with acryloyl‐β‐cyclodextrin via Michael addition enables localized anti‐inflammatory drug delivery [152]. In a recent work by Asim et al., a dithiolane‐modified gelatin system was developed that enabled photoinitiator‐free photo‐crosslinking to yield multifunctional gelatin–dithiolane (GelDT) hydrogels [153]. These hydrogels exhibited remarkable long‐term stability in culture conditions for over 28 days, supporting both encapsulated and surface‐seeded cell growth. The GelDT platform permitted pre‐gelation adjustment of mechanical strength and degradation through physical crosslinks, while post‐gelation stress‐relaxation could be modulated independently by exposure to external thiols. Additionally, GelDT facilitated covalent attachment of bioactive molecules, enabled glutathione‐triggered drug release, and showed strong suitability for 3D bioprinting owing to its shear‐thinning characteristics. The material also demonstrated excellent tissue adhesion through disulfide exchange with native thiol groups in biological tissues. Zhao et al. introduced a thiol–ene bioprinting platform based on gelatin‐norbornene (GelNB) and thiolated heparin (HepSH), offering a highly cell‐compatible and functionally versatile approach for extrusion‐based tissue fabrication [154] (Figure 7).

**FIGURE 8:**
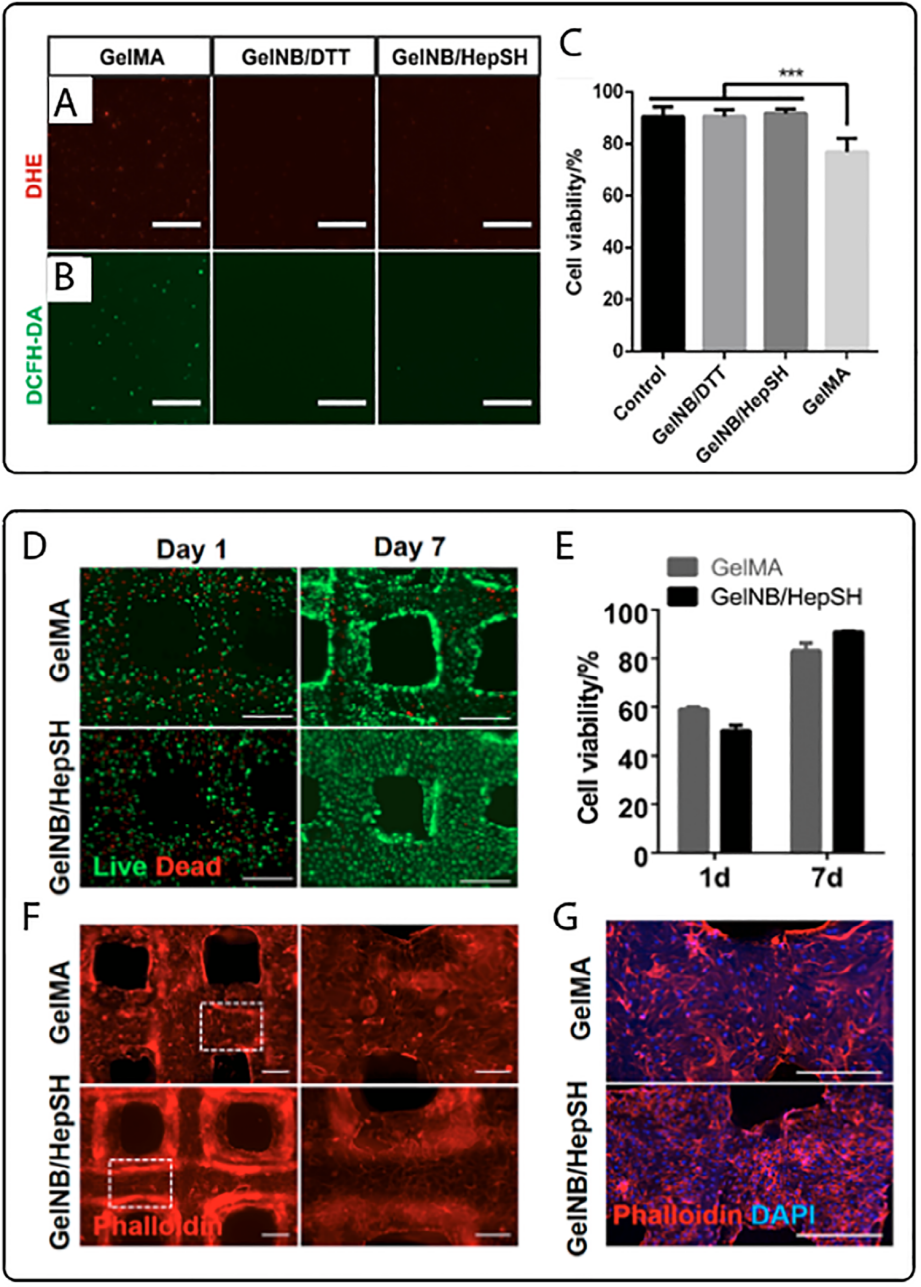
Intracellular ROS in hydrogel‐encapsulated HUVECs. HUVECs were pre‐stained with (A) DHE (red) or (B) DCFH‐DA (green) and encapsulated in GelMA, GelNB/HepSH, or GelNB/DTT hydrogels. Fluorescence images were acquired immediately after photocrosslinking (365 nm, 5 mW/cm^2^, 2 min). Scale bar: 200 µm. (C) Viability of HUVECs encapsulated in GelNB/DTT, GelNB/HepSH, and GelMA was compared with 2D controls using live/dead staining. Cell‐laden bioprinting was performed using HUVEC‐containing GelNB/HepSH (8 wt.%) and GelMA (8 wt.%) bioinks. (D) Live/dead images of bioprinted constructs at days 1 and 7; (E) corresponding viability quantification. (F and G) Cytoskeleton imaging after 7 days using inverted fluorescence and confocal microscopy (F‐actin: TRITC‐phalloidin, nuclei: DAPI). Scale bar: 500 µm. Adapted and redrawn with permission from [154]. Copyright 2021, American Chemical Society.

The resulting GelNB/HepSH hydrogels supported minimal intracellular ROS, preserved cell viability, and created pro‐angiogenic microenvironments relevant for regenerative medicine [154]. Most importantly, the bioink enabled smooth extrusion, high‐fidelity printing of complex structures, and excellent viability in encapsulated cells (Figure 7). The GelNB/HepSH hydrogel not only enabled cell laden bioprinting that was comparable to GelMA at the same gel concentration but also showed better performance in promoting growth and proliferation of encapsulated Human Umbilical Vein Endothelial Cells (HUVECs) than GelMA, mostly due to the controlled release of VEGF and ROS‐free thiol−ene crosslinking reaction (Figure 8).

**FIGURE 9:**
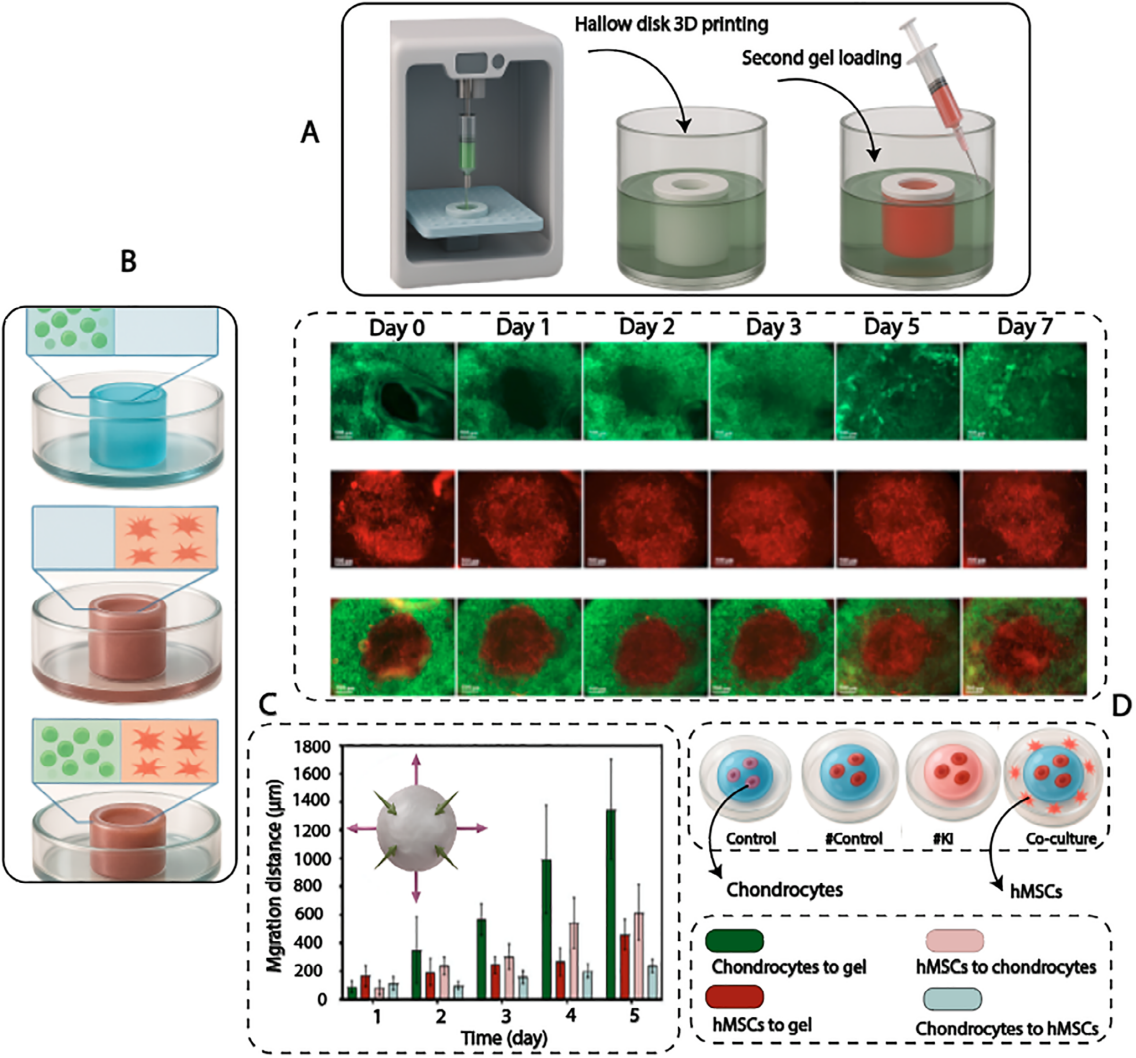
3D bioprinting of in vitro models. (A) Schematic illustration of the 3D bioprinting process to create in vitro models for studying the migration of hMSCs and chondrocytes. (B) Fluorescent microscopy images showing real‐time cell movement within the hollow cylinder with infill printed structures over 7 days. (C) Quantified migration distance of cells at different time points in the hollow cylinder with infill structures.(D) Schematic representation of the experimental groups used in the ostheoarthritis in vitro disease model. Adapted and redrawn with permission from [155]. Copyright 2026, Wiley VCH‐GmbH.

In a recent study conducted by Tavakoli et al., HA‐based bioink was designed using dynamic disulfide‐crosslinking at physiological pH by modifying HA with cysteine moieties [155]. To overcome the slow gelation kinetics typical of disulfide‐crosslinked hydrogels, potassium iodide (KI) was introduced, accelerating gelation in a concentration‐dependent manner. This enabled the fabrication of large (>3 cm) and complex 3D structures. Using this bioink, an osteoarthritis disease model was developed to investigate interactions between human mesenchymal stromal cells (hMSCs) and chondrocytes, demonstrating the immunomodulatory effect of hMSCs on inflammation‐induced chondrocytes (Figure 9).

**FIGURE 10:**
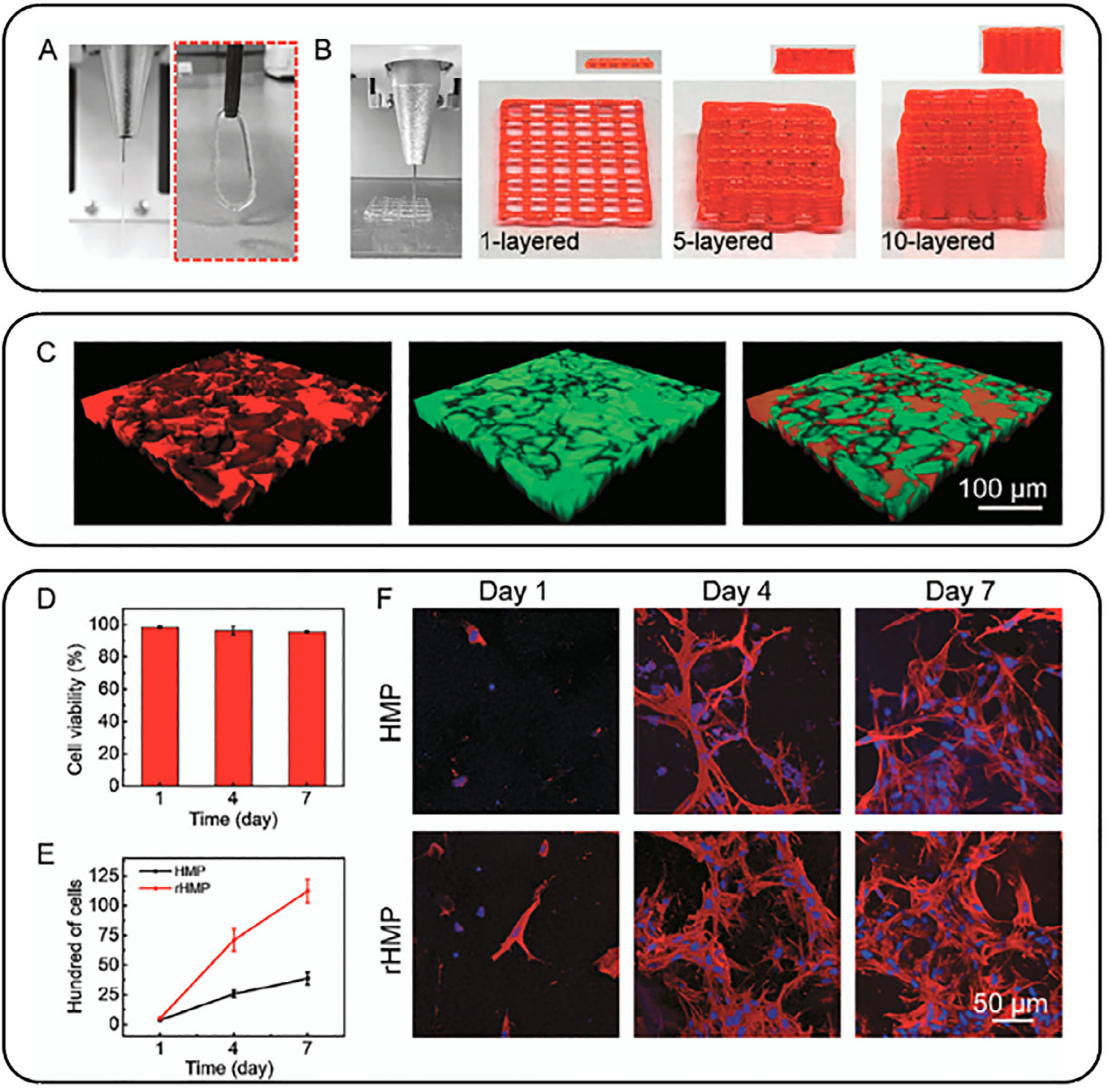
(A) The 3D printing process utilizes thiol‐modified rHMPs‐based granular hydrogels, depicted with a typical photograph of the extruded filament from a 21G conical nozzle, with the figure within the red dashed box emphasizing the material's self‐supporting properties; whereas (B) showcases a photograph of the printed grid displaying layers of deposition. The pH of the granular hydrogel ink was adjusted to 7.5 before printing. (C) 3D reconstruction shows interconnected macropores in the annealed granular hydrogel made by thiol‐modified rHMPs. (D) Cell viability at 1, 4, 7 days post‐annealing. (E) Proliferation and (F) network formation in the annealed granular hydrogel scaffold. Adapted and redrawn with permission from [156]. Copyright 2024, Wiley VCH‐GmbH.

In another study conducted by Zhang et al. functional hydrogel materials were advanced through an enzyme‐assisted surface activation strategy designed to improve the integration of non‐living and living components [156]. In this work, reactive hydrogel microparticles (HMPs) composed of thiolated hyaluronic acid and hyperbranched poly(β‐hydrazide esters) were generated with markedly enhanced surface functionality. Enzymatic activation produced more than a six‐fold increase in active group density on bulk hydrogels and a three‐fold increase on HMPs, substantially improving the accessibility of reactive motifs for post‐functionalization. This enhancement translated directly into superior initial cell adhesion and spreading, positioning the HMPs as highly effective carriers for cell culture. When integrated with 3D bioprinting, the resulting granular hydrogel ink supported robust cell adhesion, migration, proliferation, and multicellular network formation. Looking ahead, combining diverse granular hydrogel scaffolds with this enzyme‐assisted activation approach offers significant potential to enhance biological performance and broaden applications in regenerative medicine and engineered living materials (Figure 10).

## 8 Future Perspectives

Building on the expertise we have already gained, we aim to further enhance the gelation properties of thiolated polymers for 3D bioprinting. These developments will target, on the one hand, shorter gelation times and, on the other, more precise control over the gelation process, both of which are essential for printing microstructures in the range of 10–100 µm. To ensure sufficient oxygen and glucose supply and to support cell proliferation and spreading within the printed constructs, a pore size of approximately 30 µm appears crucial. Achieving such finely structured and highly interconnected pore networks will require advancements in printing technologies that go hand in hand with rapid and accurately controlled gelation of bioinks. Short gelation times are particularly important for in‐situ 3D bioprinting. This technique addresses several current limitations of conventional 3D bioprinting, as it eliminates the need for bioreactors to maintain long‐term cell viability before implantation and avoids invasive surgical procedures for construct placement [157, 158]. Instead, in‐situ 3D bioprinting enables the direct deposition of bioinks at the target site, effectively using the patient's body as a natural bioreactor to promote proper cell growth and differentiation. Integrated clinical workflow that bridges high‐resolution anatomical scanning, computed‐aided design(CAD‐based modelling), and robotic‐assisted in situ bioprinting, highlighting the potential of thiomer‐based bioinks for next‐generation regenerative procedures. Patient‐specific imaging data are transformed into detailed digital reconstructions that guide the surgeon's remote control of the robotic deposition system with high precision. The magnified inset shows thiomer bioink being extruded directly onto cardiac tissue, where rapid covalent disulfide crosslinking and strong interactions with cysteine‐containing motifs promote immediate stabilization and firm adhesion of the printed construct.

However, because surgical intervention times are limited, prolonged gelation would severely restrict the feasible size of constructs that can be printed directly into patients. Thus, reducing gelation time is key to success and can be achieved using more reactive thiomers, as described in Sections 3 and 4.

Highly reactive sulfhydryl ligands, such as mercaptobenzoic acid, are already available and can exhibit even higher reactivity when appropriately modified with neighboring functional groups. Such ligands can ensure significantly shorter gelation times; however, controlling their high reactivity remains a major technological challenge. Highly reactive thiomers often suffer from limited stability during storage and cell encapsulation. Ideally, they should remain completely inactive until extruded from the printer nozzle, followed by rapid gelation within milliseconds thereafter. Another promising direction involves the design of multifunctional thiomers that combine rapid gelation with additional properties such as strong adhesion to both encapsulated cells and target tissues, as well as controlled release of embedded bioactive compounds like growth factors or antibiotics. For instance, Han et al. developed a thiolated polymer by covalently attaching thiolactone groups to acrylamides. Upon reaction with tissue‐surface amines, the thiolactone groups release free thiols, providing strong tissue adhesion followed by crosslinking via disulfide bond formation. The resulting hydrogels demonstrated robust adhesion to various tissues alongside excellent gelation behavior [159]. Moreover, the emergence of artificial intelligence (AI), particularly deep learning techniques, has significantly advanced the capabilities of 3D bioprinting, ushering in a new generation of intelligent bioprinters [160, 161]. Equipped with specialized sensors that monitor gelation in real time, AI‐driven bioprinters will be able to precisely determine the optimal timing and conditions for thiomer gelation. Through deep learning, these systems can autonomously learn from sensor data, minimizing reliance on traditional trial‐and‐error approaches and accelerating material optimization. This data‐driven strategy will guide the design of more efficient thiolated polymers for 3D bioprinting while simultaneously reducing development costs. Additionally, supervised learning methods can be employed to model the complex relationships between thiomers and crosslinkers, enabling more accurate and reproducible printing outcomes [162]. Table 7 summarizes the key technological requirements for next‐generation thiolated polymers in 3D bioprinting, linking each challenge to corresponding material strategies and emerging solutions. By organizing these considerations into a structured overview, the table highlights how advances in thiomer chemistry and AI‐assisted optimization can drive more precise, rapid, and clinically relevant bioprinting outcomes.

**TABLE 7:** Design requirements and future directions for thiolated polymers in 3D bioprinting.

Challenge/Requirement	Key need	Potential strategies	Examples/Notes
Rapid gelation for 10–100 µm microstructures	Gelation within milliseconds	Highly reactive thiomers; electron‐withdrawing group modification	Mercaptobenzoic‐acid–based thiomers (short gelation times)
Precise gelation control	Prevent premature crosslinking	Protecting groups; triggerable thiol release; nozzle‐activated systems	Thiolactone‐modified polymers that release thiols under amine‐rich conditions
Pore size ∼30 µm for cell viability	Oxygen & nutrient diffusion; cell spreading	Tuned crosslinking density; controlled extrusion rate; microfluidic printheads	Essential for long‐term HUVEC proliferation
Stability during storage and encapsulation	Prevent early oxidation/aggregation	Freeze‐drying, inert packaging, storage at low oxygen tension	Challenge for highly reactive thiomers
In‐situ 3D bioprinting applications	Very short surgical time window	Fast on‐demand gelation; body as natural bioreactor	Eliminates bioreactors and invasive implantation
Multifunctionality	Adhesion + gelation + drug delivery	Combine adhesive motifs, thiolactones, and controlled‐release moieties	Han et al. thiolactone–acrylamide polymer (strong tissue adhesion + crosslinking)
AI‐guided optimization	Real‐time gelation monitoring	Sensor‐equipped printers; deep learning for gelation prediction	Data‐driven design of thiomers; reduced trial‐and‐error
Reproducible bonding behavior	Predictable thiomer–crosslinker interaction	Supervised learning to model reaction pathways	Enables standardization across printers

## 9 Conclusion

Compared with other polymers used as extracellular matrix (ECM) materials in 3D bioprinting, thiomers exhibit the broadest spectrum of crosslinking reactivities. This exceptional versatility enables the development of diverse bioinks with precisely tunable gelation behavior, including dual‐stage and hybrid crosslinking mechanisms. Because disulfide bonds, one of nature's most fundamental structural linkages, can form not only through oxidation but also via thiol–disulfide exchange reactions, thiomer‐based networks display self‐healing properties that promote seamless integration between printed layers. Furthermore, their ability to form covalent disulfide bonds with cysteine‐containing motifs in encapsulated cells and tissue components confers strong bioadhesive characteristics, making thiomers particularly promising for in situ 3D bioprinting applications. To appreciate their potential, one might simply envision protein chemistry with and without cysteine, the presence of this single amino acid transforms structural dynamics dramatically. Similarly, thiomers, with their unique chemical adaptability and biological compatibility, are poised to reshape the landscape of 3D bioprinting, driving the field toward more efficient and functionally advanced therapeutic systems.

## Funding

No external funding was received for this study.

## Conflicts of Interest

The authors declare no conflicts of interest.

## Data Availability

Data sharing does not apply to this article.

## References

[1] A. C. Sousa , R. Alvites , B. Lopes , et al., “Three‐Dimensional Printing/Bioprinting and Cellular Therapies for Regenerative Medicine: Current Advances,” Journal of Functional Biomaterials 16, no. 1 (2025): 28, 10.3390/jfb16010028. 39852584 PMC11765675

[2] M. Rama , A. S. Suresh , A. E. John , L. Vijaylal , and U. Vijayalakshmi , “Revolutionizing Tissue Engineering: Integrating 3D Bioprinting and Additive Manufacturing for Precision Scaffold Design and Biomimetic Tissue Regeneration,” Materials Technology 40, no. 1 (2025): 2530639, 10.1080/10667857.2025.2530639.

[3] S. Singh , M. Kumar , D. Kumar , S. Kumar , S. Chopra , and A. Bhatia , “Therapeutic Precision: Unveiling the Potential of 3D Printing in Drug Delivery, Tissue Engineering, and Regenerative Medicine,” 3D Printing and Additive Manufacturing 12 (2024): 447–473, 10.1089/3dp.2023.0364. PMC12670717 41341005

[4] R. Levato , T. Jungst , R. G. Scheuring , T. Blunk , J. Groll , and J. Malda , “From Shape to Function: The Next Step in Bioprinting,” Advanced Materials 32, no. 12 (2020): 1906423, 10.1002/adma.201906423. PMC7116209 32045053

[5] I. Donderwinkel , J. C. M. Van Hest , and N. R. Cameron , “Bio‐Inks for 3D Bioprinting: Recent Advances and Future Prospects,” Polymer Chemistry 8, no. 31 (2017): 4451–4471, 10.1039/C7PY00826K.

[6] A. Schwab , R. Levato , M. D'este , S. Piluso , D. Eglin , and J. Malda , “Printability and Shape Fidelity of Bioinks in 3D Bioprinting,” Chemical Reviews 120, no. 19 (2020): 11028–11055. 32856892 10.1021/acs.chemrev.0c00084 PMC7564085

[7] H. W. Ooi , C. Mota , A. T. Ten Cate , A. Calore , L. Moroni , and M. B. Baker , “Thiol–Ene Alginate Hydrogels as Versatile Bioinks for Bioprinting,” Biomacromolecules 19, no. 8 (2018): 3390–3400, 10.1021/acs.biomac.8b00696. 29939754 PMC6588269

[8] J. Gopinathan and I. Noh , “Click Chemistry‐Based Injectable Hydrogels and Bioprinting Inks for Tissue Engineering Applications,” Tissue Engineering and Regenerative Medicine 15, no. 5 (2018): 531–546, 10.1007/s13770-018-0152-8. 30603577 PMC6171698

[9] A. Bernkop‐Schnürch , “Thiomers: A New Generation of Mucoadhesive Polymers,” Advanced Drug Delivery Reviews 57, no. 11 (2005): 1569–1582, 10.1016/j.addr.2005.07.002. 16176846

[10] S. Haddadzadegan , S. Summonte , F. Ricci , M. Sandmeier , and A. Bernkop‐Schnürch , “Intraoral Drug Delivery: Bridging the Gap between Academic Research and Industrial Innovations,” Advanced Functional Materials 35 (2025): 2500157, 10.1002/adfm.202500157.

[11] G. Kali , A. M. Taha , and E. Campanella , “Enhanced Mucoadhesion of Thiolated β‐Cyclodextrin by S‐Protection With 2‐Mercaptoethanesulfonic Acid,” ACS Omega 9 (2024): 5819–5828. 38343993 10.1021/acsomega.3c08836 PMC10851230

[12] P. Knoll , N.‐M. Le , R. Wibel , et al., “Thiolated Pectins: In Vitro and Ex Vivo Evaluation of Three Generations of Thiomers,” Acta Biomaterialia 135 (2021): 139–149, 10.1016/j.actbio.2021.08.016. 34418540

[13] A. Fürst , G. Kali , N. A. Efiana , Z. B. Akkuş‐Dağdeviren , S. Haddadzadegan , and A. Bernkop‐Schnürch , “Thiolated Cyclodextrins: A Comparative Study of Their Mucoadhesive Properties,” International Journal of Pharmaceutics 635 (2023): 122719. 36791998 10.1016/j.ijpharm.2023.122719

[14] S. Haddadzadegan , P. Knoll , R. Wibel , G. Kali , and A. Bernkop‐Schnürch , “Three Generations of Thiolated Cyclodextrins: A Direct Comparison of Their Mucus Permeating and Mucoadhesive Properties,” Acta Biomaterialia 167 (2023): 309–320, 10.1016/j.actbio.2023.05.050. 37271247

[15] S. Haddadzadegan , A. Saleh , F. Veider , et al., “Cyclodextrin‐Mediated Enhancement of Gastrointestinal Drug Delivery: Unveiling Mucoadhesive and Mucopenetrating Synergy,” Drug Delivery and Translational Research 15 (2025): 1–15. 40113660 10.1007/s13346-025-01832-w PMC12397179

[16] F. Veider , S. Haddadzadegan , E. S. Armengol , F. Laffleur , G. Kali , and A. Bernkop‐Schnürch , “Inhibition of P‐Glycoprotein‐Mediated Efflux by Thiolated Cyclodextrins,” Carbohydrate Polymers 327 (2024): 121648. 38171673 10.1016/j.carbpol.2023.121648

[17] D. To , G. Kali , S. Haddadzadegan , et al., “Power‐Up for Mucoadhesiveness: Two Generations of Thiolated Surfactants for Enhanced Sticky Nanoemulsions,” ACS Biomaterials Science & Engineering 9, no. 12 (2023): 6797–6804, 10.1021/acsbiomaterials.3c01207. 37996083 PMC10716821

[18] R. A. Khan , S. Arshad , H. Sadiac , and A. A. Naseemd , “Emerging Trend of Thiolated Polymers/Materials and Nanomedicine in Wound Healing,” Global Pharmaceutical Sciences Review 6, no. 1 (2021): 36–54, 10.31703/gpsr.2021(VI-I).05.

[19] M. Chrószcz‐Porębska and A. Gadomska‐Gajadhur , “Cysteine Conjugation: An Approach to Obtain Polymers with Enhanced Muco‐and Tissue Adhesion,” International Journal of Molecular Sciences 25 (2024): 12177. 39596243 10.3390/ijms252212177 PMC11594736

[20] B. Le‐Vinh , C. Steinbring , N.‐M. Nguyen Le , B. Matuszczak , and A. Bernkop‐Schnurch , “S‐Protected Thiolated Chitosan Versus Thiolated Chitosan as Cell Adhesive Biomaterials for Tissue Engineering,” ACS Applied Materials & Interfaces 15, no. 34 (2023): 40304–40316, 10.1021/acsami.3c09337. 37594415 PMC10472333

[21] S. Noreen and A. Bernkop‐Schnürch , “Thiolated Poly‐ and Oligosaccharide‐Based Hydrogels for Tissue Engineering and Wound Healing,” Advanced Functional Materials 34, no. 4 (2024): 2310129, 10.1002/adfm.202310129.

[22] C. E. Kast , W. Frick , U. Losert , and A. Bernkop‐Schnürch , “Chitosan‐Thioglycolic Acid Conjugate: A New Scaffold Material for Tissue Engineering?,” International Journal of Pharmaceutics 256 (2003): 183–189, 10.1016/S0378-5173(03)00076-0. 12695025

[23] M. Aghajani , H. R. Garshasbi , S. M. Naghib , and M. R. Mozafari , “3D Printing of Hydrogel Polysaccharides for Biomedical Applications: A Review,” Biomedicines 13, no. 3 (2025): 731, 10.3390/biomedicines13030731. 40149707 PMC11940176

[24] T. Beran , T. Mulholland , F. Henning , N. Rudolph , and T. A. Osswald , “Nozzle Clogging Factors During Fused Filament Fabrication of Spherical Particle Filled Polymers,” Additive Manufacturing 23 (2018), 206–214.

[25] D. X. B. Chen , “Extrusion Bioprinting of Scaffolds,” in Extrusion Bioprinting of Scaffolds for Tissue Engineering Applications (Springer, 2018), 117–145.

[26] L. Ning and X. Chen , “A Brief Review of Extrusion‐Based Tissue Scaffold Bio‐Printing,” Biotechnology Journal 12, no. 8 (2017): 1600671, 10.1002/biot.201600671. 28544779

[27] Z. Davoudi , G. Kali , D. Braun , M. H. Azizi , and A. Bernkop‐Schnürch , “Highly Thiolated Corn Starch for Enhanced Mucoadhesion and Permeation,” International Journal of Pharmaceutics 680 (2025): 125798, 10.1016/j.ijpharm.2025.125798. 40446874

[28] F. Hintzen , F. Laffleur , F. Sarti , G. Shahnaz , and A. Bernkop‐Schnürch , “Thiomers: Influence of Molar Mass on In Situ Gelling Properties,” International Journal of Pharmaceutics 436, no. 1–2 (2012): 120–126, 10.1016/j.ijpharm.2012.05.073. 22683454

[29] K. S. Jensen , R. E. Hansen , and J. R. Winther , “Kinetic and Thermodynamic Aspects of Cellular Thiol–Disulfide Redox Regulation,” Antioxidants & redox signaling 11, no. 5 (2009): 1047–1058, 10.1089/ars.2008.2297. 19014315

[30] Y. Zheng , W. Zheng , D. Zhu , and H. Chang , “Theoretical Modeling of p Ka 's of Thiol Compounds in Aqueous Solution,” New Journal of Chemistry 43, no. 13 (2019): 5239–5254, 10.1039/C8NJ06259E.

[31] D. H. A. Bianchi , “The Paradoxical Influence of the pKa on the Reactivity of Thiols and Its Biological Relevance,” MaRBLe 2 (2014): 222–231.

[32] C. Chu , D. Stamatelatos , and K. McNeill , “Aquatic Indirect Photochemical Transformations of Natural Peptidic Thiols: Impact of Thiol Properties, Solution pH, Solution Salinity and Metal Ions,” Environmental Science: Processes & Impacts 19, no. 12 (2017): 1518–1527. 29090717 10.1039/c7em00324b

[33] L. Polgár , “On the Mode of Activation of the Catalytically Essential Sulfhydryl Group of Papain,” European Journal of Biochemistry 33, no. 1 (1973): 104–109, 10.1111/j.1432-1033.1973.tb02660.x. 4691346

[34] A. Cstorer and R. Ménard , “[33]Catalytic Mechanism in Papain family of Cysteine peptidases,” in Methods in Enzymology (Elsevier, 1994), 486–500. 10.1016/0076-6879(94)44035-2 7845227

[35] D. Bermejo‐Velasco , A. Azémar , O. P. Oommen , J. Hilborn , and O. P. Varghese , “Modulating Thiol p K a Promotes Disulfide Formation at Physiological pH: An Elegant Strategy to Design Disulfide Cross‐Linked Hyaluronic Acid Hydrogels,” Biomacromolecules 20, no. 3 (2019): 1412–1420, 10.1021/acs.biomac.8b01830. 30726668

[36] P. Nagy , “Kinetics and Mechanisms of Thiol–Disulfide Exchange Covering Direct Substitution and Thiol Oxidation‐Mediated Pathways,” Antioxidants & Redox Signaling 18, no. 13 (2013): 1623–1641, 10.1089/ars.2012.4973. 23075118 PMC3613173

[37] C. Menzel , J. Silbernagl , F. Laffleur , et al., “2,2′Dithiodinicotinyl Ligands: Key to More Reactive Thiomers,” International Journal of Pharmaceutics 503, no. 1–2 (2016): 199–206, 10.1016/j.ijpharm.2016.03.010. 26972378

[38] P. A. Fernandes and M. J. Ramos , “Theoretical Insights Into the Mechanism for Thiol/Disulfide Exchange,” Chemistry–A European Journal 10, no. 1 (2004): 257–266. 14695571 10.1002/chem.200305343

[39] D. M. Beaupre and R. G. Weiss , “Thiol‐and Disulfide‐Based Stimulus‐responsive Soft Materials and Self‐Assembling Systems,” Molecules 26, no. 11 (2021): 3332, 10.3390/molecules26113332. 34206043 PMC8199128

[40] G. H. Snyder , M. J. Cennerazzo , A. J. Karalis , and D. Locey , “Electrostatic Influence of Local Cysteine Environments on Disulfide Exchange Kinetics,” Biochemistry 20, no. 23 (1981): 6509–6519, 10.1021/bi00526a001. 6796114

[41] L. B. Poole , “The Basics of Thiols and Cysteines in Redox Biology and Chemistry,” Free Radical Biology and Medicine 80 (2015): 148–157, 10.1016/j.freeradbiomed.2014.11.013. 25433365 PMC4355186

[42] S. Summonte , G. F. Racaniello , A. Lopedota , N. Denora , and A. Bernkop‐Schnürch , “Thiolated Polymeric Hydrogels for Biomedical Application: Cross‐linking Mechanisms,” Journal of Controlled Release 330 (2021): 470–482, 10.1016/J.JCONREL.2020.12.037. 33359581

[43] A. H. Krauland , M. H. Hoffer , and A. Bernkop‐Schnürch , “Viscoelastic Properties of a New In Situ Gelling Thiolated Chitosan Conjugate,” Drug Development and Industrial Pharmacy 31, no. 9 (2005): 885–893. 16306000 10.1080/03639040500271985

[44] D. Sakloetsakun , J. M. R. Hombach , and A. Bernkop‐Schnürch , “In Situ Gelling Properties of Chitosan‐Thioglycolic Acid Conjugate in the Presence of Oxidizing Agents,” Biomaterials 30, no. 31 (2009): 6151–6157, 10.1016/j.biomaterials.2009.07.060. 19699516

[45] M. H. Asim , S. Silberhumer , I. Shahzadi , A. Jalil , B. Matuszczak , and A. Bernkop‐Schnürch , “S‐Protected Thiolated Hyaluronic Acid: In‐Situ Crosslinking Hydrogels for 3D Cell Culture Scaffold,” Carbohydrate Polymers 237 (2020): 116092. 32241444 10.1016/j.carbpol.2020.116092

[46] A. Aerts , M. Vovchenko , S. A. Elahi , et al., “A Spontaneous In Situ Thiol‐ene Crosslinking Hydrogel With Thermo‐Responsive Mechanical Properties,” Polymers 16, no. 9 (2024): 1264, 10.3390/polym16091264. 38732733 PMC11085619

[47] B. H. Lee , B. West , R. McLemore , C. Pauken , and B. L. Vernon , “In‐Situ Injectable Physically and Chemically Gelling NIPAAm‐Based Copolymer System for Embolization,” Biomacromolecules 7, no. 6 (2006): 2059–2064, 10.1021/bm060211h. 16768434 PMC2653053

[48] V. Cheng , B. H. Lee , C. Pauken , and B. L. Vernon , “Poly( N ‐Isopropylacrylamide‐ Co ‐Poly(Ethylene Glycol))‐Acrylate Simultaneously Physically and Chemically Gelling Polymer Systems,” Journal of Applied Polymer Science 106, no. 2 (2007): 1201–1207, 10.1002/app.26760.

[49] S. A. Robb , B. H. Lee , R. McLemore , and B. L. Vernon , “Simultaneously Physically and Chemically Gelling Polymer System Utilizing a Poly (NIPAAm‐co‐cysteamine)‐Based Copolymer,” Biomacromolecules 8, no. 7 (2007): 2294–2300, 10.1021/bm070267r. 17567067 PMC2892919

[50] S. Stichler , T. Jungst , M. Schamel , et al., “Thiol‐ene Clickable Poly (glycidol) Hydrogels for Biofabrication,” Annals of biomedical engineering 45, no. 1 (2017): 273–285, 10.1007/s10439-016-1633-3. 27177637

[51] T. B. Dorsey , A. Grath , A. Wang , C. Xu , Y. Hong , and G. Dai , “Evaluation of Photochemistry Reaction Kinetics to Pattern Bioactive Proteins on Hydrogels for Biological Applications,” Bioactive materials 3, no. 1 (2018): 64–73, 10.1016/j.bioactmat.2017.05.005. 29632897 PMC5889137

[52] Y. Zhong , X. Zhao , G. Li , D. Zhang , and D. Wang , “Mussel‐inspired Hydrogels as Tissue Adhesives for Hemostasis With Fast‐forming and Self‐healing Properties,” European Polymer Journal 148 (2021): 110361, 10.1016/j.eurpolymj.2021.110361.

[53] J. H. Ryu , Y. Lee , W. H. Kong , T. G. Kim , T. G. Park , and H. Lee , “Catechol‐Functionalized Chitosan/Pluronic Hydrogels for Tissue Adhesives and Hemostatic Materials,” Biomacromolecules 12, no. 7 (2011): 2653–2659, 10.1021/bm200464x. 21599012

[54] Z. Zeng and X. Mo , “Rapid In Situ Cross‐Linking of Hydrogel Adhesives Based on Thiol‐Grafted Bio‐Inspired Catechol‐Conjugated Chitosan,” Journal of Biomaterials Applications 32, no. 5 (2017): 612–621, 10.1177/0885328217738403. 29113567

[55] C. Leichner , M. Jelkmann , and A. Bernkop‐Schnürch , “Thiolated Polymers: Bioinspired Polymers Utilizing One of the Most Important Bridging Structures in Nature,” Advanced Drug Delivery Reviews 151–152 (2019): 191–221, 10.1016/j.addr.2019.04.007. 31028759

[56] A. Dicks and C. Woolard , “Thiol‐X Chemistry: A Skeleton Key Unlocking Advanced Polymers in Additive Manufacturing,” Macromolecular Materials and Engineering 310 (2025): 2400445, 10.1002/mame.202400445.

[57] K. Ghosh , X. Z. Shu , R. Mou , et al., “Rheological Characterization of In Situ Cross‐linkable Hyaluronan Hydrogels,” Biomacromolecules 6, no. 5 (2005): 2857–2865, 10.1021/bm050361c. 16153128

[58] H. Mutlu , E. B. Ceper , X. Li , et al., “Sulfur Chemistry in Polymer and Materials Science,” Macromolecular rapid communications 40 (2019): 1800650, 10.1002/marc.201800650. 30468540

[59] A. B. Lowe , “Thiol‐ene “Click” Reactions and Recent Applications in Polymer and Materials Synthesis,” Polymer Chemistry 1, no. 1 (2010): 17–36, 10.1039/B9PY00216B.

[60] P. Wadhwa , A. Kharbanda , and A. T.‐M. Sharma , “Thia‐Michael Addition: An Emerging Strategy in Organic Synthesis,” Asian Journal of Organic Chemistry 7, no. 4 (2018): 634–661, 10.1002/ajoc.201700609.

[61] D. P. Nair , M. Podgórski , S. Chatani , et al., “The Thiol‐Michael Addition Click Reaction: A Powerful and Widely Used Tool in Materials Chemistry,” Chemistry of Materials 26, no. 1 (2014): 724–744, 10.1021/cm402180t.

[62] J. W. Chan , C. E. Hoyle , A. B. Lowe , and M. Bowman , “Nucleophile‐Initiated Thiol‐Michael Reactions: Effect of Organocatalyst, Thiol, and Ene,” Macromolecules 43, no. 15 (2010): 6381–6388, 10.1021/ma101069c.

[63] J. Su , “Thiol‐Mediated Chemoselective Strategies for In Situ Formation of Hydrogels,” Gels 4, no. 3 (2018): 72, 10.3390/gels4030072. 30674848 PMC6209259

[64] B. D. Fairbanks , T. F. Scott , C. J. Kloxin , K. S. Anseth , and C. N. Bowman , “Thiol− Yne Photopolymerizations: Novel Mechanism, Kinetics, and Step‐Growth Formation of Highly Cross‐Linked Networks,” Macromolecules 42, no. 1 (2009): 211–217, 10.1021/ma801903w. 19461871 PMC2651690

[65] J. C. Worch , C. J. Stubbs , M. J. Price , and A. P. Dove , “Click Nucleophilic Conjugate Additions to Activated Alkynes: Exploring Thiol‐yne, Amino‐yne, and Hydroxyl‐yne Reactions From (bio) Organic to Polymer Chemistry,” Chemical Reviews 121, no. 12 (2021): 6744–6776, 10.1021/acs.chemrev.0c01076. 33764739 PMC8227514

[66] B. D. Fairbanks , E. A. Sims , K. S. Anseth , and C. N. Bowman , “Reaction Rates and Mechanisms for Radical, Photoinitated Addition of Thiols to Alkynes, and Implications for Thiol− Yne Photopolymerizations and Click Reactions,” Macromolecules 43, no. 9 (2010): 4113–4119, 10.1021/ma1002968.

[67] M. D. Ramírez‐Alba , L. Resina , J. García‐Torres , R. Macovez , C. Alemán , and M. M. Pérez‐Madrigal , “Thiol‐yne Crosslinked Alginate Click‐Hydrogel for the Electrical Stimulation of Skin Wound Healing,” International Journal of Biological Macromolecules 322 (2025): 146880, 10.1016/j.ijbiomac.2025.146880. 40818701

[68] Z. Xia , B. Zhao , J. Xiang , et al., “Injectable pH‐Responsive Hydrogel Adapted to Gingival Crevicular Fluid Microenvironment for Periodontitis Therapy,” ACS Applied Materials & Interfaces 17, no. 21 (2025): 31357–31367, 10.1021/acsami.5c02776. 40383914

[69] S. Li , J. Chen , J. Wang , and H. Zeng , “Anti‐Biofouling Materials and Surfaces Based on Mussel‐Inspired Chemistry,” Materials Advances 2 (2021): 2216–2230, 10.1039/D1MA00053E.

[70] L. K Jang , J. T Ahlquist , C. Ye , et al., “Rapid Curing Dynamics of PEG‐thiol‐ene Resins Allow Facile 3D Bioprinting and in‐Air Cell‐Laden Microgel Fabrication,” Biomedical Materials 20, no. 1 (2025): 015009, 10.1088/1748-605X/ad8540. 39584565

[71] E. Maloney , C. Clark , H. Sivakumar , et al., “Immersion Bioprinting of Tumor Organoids in Multi‐Well Plates for Increasing Chemotherapy Screening Throughput,” Micromachines 11, no. 2 (2020): 208, 10.3390/mi11020208. 32085455 PMC7074680

[72] P. Puistola , Novel Bioink Design for 3d Bioprinting of Human Pluripotent Stem Cell Derived Corneal Epithelial Cells (Tampere University, 2020).

[73] C. García‐Astrain , M. Henriksen‐Lacey , E. Lenzi , et al., “A Scaffold‐Assisted 3D Cancer Cell Model for Surface‐Enhanced Raman Scattering‐Based Real‐Time Sensing and Imaging,” ACS nano 18, no. 17 (2024): 11257–11269, 10.1021/acsnano.4c00543. 38632933 PMC11064228

[74] K. Al‐Husaini , E. Spessot , E. Baena , M. Domingos , and A. Tirella , “Bio‐Fabricated Alginate Tumor‐Like Hydrogels to Enhance Understanding of Prostate‐Specific Micro‐Environments In Vitro,” BioRxiv (2025): 2025–2028.

[75] Z. Gao , S. Ding , T. Fan , et al., “Protocol for Embedded 3D Printing of Heart Tissues Using Thiol‐Norbornene Collagen,” STAR protocols 5, no. 2 (2024): 102994, 10.1016/j.xpro.2024.102994. 38568815 PMC10999940

[76] J. Fernández‐Pérez , A. Seijas‐Gamardo , A. J. Feliciano , et al., “Highly Tunable and Cell‐Remodelable Thiol‐ene Alginate‐Peptide Crosslinked Hydrogels to Recreate Cellular and Organoid Microenvironments for Biofabrication,” Advanced Healthcare Materials 15 (2026): 04313, 10.1002/adhm.202504313. PMC12973339 41334632

[77] M. Jankowska , O. Ozukanar , E. Çakmakçi , and J. Ortyl , “A Thiol‐Functional Amine Synergist as a Co‐Initiator for DLP 3D Printing Applications,” Polymer Chemistry 16, no. 35 (2025): 3895–3915, 10.1039/D5PY00603A.

[78] L. Troncoso‐Afonso , Y. M. Henríquez‐Banegas , G. A. Vinnacombe‐Willson , et al., “Using Thiol–Ene Click Chemistry to Engineer 3D Printed Plasmonic Hydrogel Scaffolds for SERS Biosensing,” Biomaterials Science 13, no. 11 (2025): 2936–2950, 10.1039/D4BM01529K. 40237173 PMC12001321

[79] P. A. Amorim , M. A. Ávila , R. Anand , P. Moldenaers , P. Van Puyvelde , and V. Bloemen , “Insights on Shear Rheology of Inks for Extrusion‐Based 3D Bioprinting,” Bioprinting 22 (2021): e00129, 10.1016/j.bprint.2021.e00129.

[80] H. Herrada‐Manchón , M. A. Fernández , and E. Aguilar , “Essential Guide to Hydrogel Rheology in Extrusion 3D Printing: How to Measure it and Why it Matters?,” Gels 9, no. 7 (2023): 517. 37504396 10.3390/gels9070517 PMC10379134

[81] S. C. Lee , G. Gillispie , P. Prim , and S. J. Lee , “Physical and Chemical Factors Influencing the Printability of Hydrogel‐Based Extrusion Bioinks,” Chemical reviews 120, no. 19 (2020): 10834–10886, 10.1021/acs.chemrev.0c00015. 32815369 PMC7673205

[82] A. Kjar , B. McFarland , K. Mecham , N. Harward , and Y. Huang , “Engineering of Tissue Constructs Using Coaxial Bioprinting,” Bioactive materials 6, no. 2 (2021): 460–471, 10.1016/j.bioactmat.2020.08.020. 32995673 PMC7490764

[83] W. Li , J. Li , C. Pan , J.‐S. Lee , B. S. Kim , and G. Gao , “Light‐Based 3D Bioprinting Techniques for Illuminating the Advances of Vascular Tissue Engineering,” Materials Today Bio 29 (2024): 101286, 10.1016/j.mtbio.2024.101286. PMC11492625 39435375

[84] T. Steudter , T. Lam , H. Pirmahboub , et al., “Hyaluronic Acid‐Based Inks for Stereolithography (Bio) Printing: Benefits of Thiol‐ene Vs,” ChemRxiv (2024).

[85] H. Kumar and K. Kim , “Stereolithography 3D Bioprinting,” in 3D Bioprinting: Principles and protocols (Springer, 2020), 93–108. 10.1007/978-1-0716-0520-2_6 32207107

[86] R. Levato , O. Dudaryeva , C. E. Garciamendez‐Mijares , et al., “Light‐Based Vat‐Polymerization Bioprinting,” Nature Reviews Methods Primers 3, no. 1 (2023): 47, 10.1038/s43586-023-00231-0. PMC12407150 40909236

[87] M. Zanon , L. Montalvillo‐Jiménez , R. Cue‐López , et al., “Vat 3D Printing of Full‐Alginate Hydrogels Via Thiol–Ene Reactions Towards Tissue Engineering Applications,” Polymer Chemistry 14, no. 42 (2023): 4856–4868, 10.1039/D3PY00902E.

[88] E. Rossegger , Y. Li , H. Frommwald , and S. Schlögl , “Vat Photopolymerization 3D Printing With Light‐Responsive Thiol‐Norbornene Photopolymers,” Monatshefte für Chemie—Chemical Monthly 154, no. 5 (2023): 473–480, 10.1007/s00706-022-03016-5.

[89] B. D. Fairbanks , M. P. Schwartz , A. E. Halevi , C. R. Nuttelman , C. N. Bowman , and K. S. Anseth , “A Versatile Synthetic Extracellular Matrix Mimic via Thiol‐Norbornene Photopolymerization,” Advanced Materials 21 (2009): 5005. 25377720 10.1002/adma.200901808 PMC4226179

[90] U. Arickx , Fabrication of Highly Robust Thiol‐Ene Click Chemistry‐Based Hydrogel Scaffold for 3D DLP Printing, Master's Thesis, Maastricht University, Maastricht, The Netherlands (2023).

[91] S. Pal and S. K. Asha , “Thiol‐Ene‐Based Degradable 3D Printed Network From Bio Resource Derived Monomers Ethyl‐Lactate and Isosorbide,” European Polymer Journal 205 (2024): 112761, 10.1016/j.eurpolymj.2024.112761.

[92] A. Cem and B. Şenol , “Advances in Digital Light Processing (DLP) Bioprinting: A Review of Biomaterials and Its Applications, Innovations, Challenges, and Future Perspectives,” Polymers 17, no. 9 (2025): 1287. 40363070 10.3390/polym17091287 PMC12074245

[93] W. Li , M. Wang , H. Ma , F. A. Chapa‐Villarreal , A. O. Lobo , and Y. S. Zhang , “Stereolithography Apparatus and Digital Light Processing‐based 3D Bioprinting for Tissue Fabrication,” Iscience 26, no. 2 (2023): 106039, 10.1016/j.isci.2023.106039. 36761021 PMC9906021

[94] L. Nie , Y. Sun , O. V. Okoro , Y. Deng , G. Jiang , and A. Shavandi , “Click Chemistry for 3D Bioprinting,” Materials Horizons 10, no. 8 (2023): 2727–2763, 10.1039/D3MH00516J. 37170645

[95] Y. Luo , W. Sun , M. Bao , et al., “Process Fundamentals and Quality Investigation in Extrusion 3D Printing of Shear Thinning Materials: Extrusion Process Based on Nishihara Model,” The International Journal of Advanced Manufacturing Technology 124, no. 1 (2023): 245–264, 10.1007/s00170-022-10506-7.

[96] M. Brillinger and K. A. Pendl , “Non Newtonian Material Behaviour in Extrusion Based 3D Printing: Investigation of Critical Process Parameters,” Paper presented at Proceedings of the 6th World Congress on Mechanical, Chemical, and Material Engineering , August 2020, 1–10.

[97] G. A. Campbell , M. E. Zak , and M. D. Wetzel , “Newtonian, Power Law, and Infinite Shear Flow Characteristics of Concentrated Slurries Using Percolation Theory Concepts,” Rheologica Acta 57, no. 3 (2018): 197–216, 10.1007/s00397-017-1070-8.

[98] S. Ansari , M. A. I. Rashid , and P. R. Waghmare , “Measurement of the Flow Behavior Index of Newtonian and Shear‐Thinning Fluids via Analysis of the Flow Velocity Characteristics in a Mini‐Channel,” SN Applied Sciences 2, no. 11 (2020): 1787, 10.1007/s42452-020-03561-w.

[99] S. Kyle , Z. M. Jessop , A. Al‐Sabah , and I. S. Whitaker , “Printability' of Candidate Biomaterials for Extrusion Based 3D Printing: State‐of‐the‐Art,” Advanced Healthcare Materials 6 (2017): 1700264, 10.1002/adhm.201700264. 28558161

[100] J. Wu , C. Wu , S. Zou , et al., “Investigation of Biomaterial Ink Viscosity Properties and Optimization of the Printing Process Based on Pattern Path Planning,” Bioengineering 10, no. 12 (2023): 1358, 10.3390/bioengineering10121358. 38135949 PMC10740413

[101] G. Stojkov , Z. Niyazov , F. Picchioni , and R. K. Bose , “Relationship Between Structure and Rheology of Hydrogels for Various Applications,” Gels 7, no. 4 (2021): 255, 10.3390/gels7040255. 34940315 PMC8700820

[102] M. Bercea , “Rheology as a Tool for Fine‐Tuning the Properties of Printable Bioinspired Gels,” Molecules 28, no. 6 (2023): 2766, 10.3390/molecules28062766. 36985738 PMC10058016

[103] P. Wei , C. Cipriani , C.‐M. Hsieh , K. Kamani , S. Rogers , and E. Pentzer , “Go With the Flow: Rheological Requirements for Direct Ink Write Printability,” Journal of Applied Physics 134, no. 10 (2023): 1–39.

[104] R. Holmes , X. B. Yang , A. Dunne , L. Florea , D. Wood , and G. Tronci , “Thiol‐ene Photo‐Click Collagen‐PEG Hydrogels: Impact of Water‐Soluble Photoinitiators on Cell Viability, Gelation Kinetics and Rheological Properties,” Polymers 9, no. 6 (2017): 226, 10.3390/polym9060226. 30970903 PMC6431953

[105] S. M. Hull , Crosslinking Strategies for 3D Bioprinting of Engineered Hydrogels (Stanford University, 2022).

[106] C. A. DeForest , B. D. Polizzotti , and K. S. Anseth , “Sequential Click Reactions for Synthesizing and Patterning Three‐Dimensional Cell Microenvironments,” Nature materials 8, no. 8 (2009): 659–664, 10.1038/nmat2473. 19543279 PMC2715445

[107] Z. Mũnoz , H. Shih , and C.‐C. Lin , “Gelatin Hydrogels Formed by Orthogonal Thiol–Norbornene Photochemistry for Cell Encapsulation,” Biomaterials Science 2, no. 8 (2014): 1063–1072, 10.1039/C4BM00070F. 32482001

[108] B. H. Northrop and R. N. Coffey , “Thiol–Ene Click Chemistry: Computational and Kinetic Analysis of the Influence of Alkene Functionality,” Journal of the American Chemical Society 134, no. 33 (2012): 13804–13817, 10.1021/ja305441d. 22853003

[109] T. Göckler , S. Haase , X. Kempter , et al., “Tuning Superfast Curing Thiol‐Norbornene‐Functionalized Gelatin Hydrogels for 3D Bioprinting,” Advanced Healthcare Materials 10 (2021): 2100206. 34145799 10.1002/adhm.202100206 PMC11481056

[110] C. R. Fellin and A. Nelson , “Direct‐Ink Write 3D Printing Multistimuli‐Responsive Hydrogels and Post‐Functionalization via Disulfide Exchange,” ACS Applied Polymer Materials 4, no. 5 (2022): 3054–3061, 10.1021/acsapm.1c01538. 38239328 PMC10795753

[111] T. S. Mohan , P. Datta , S. Nesaei , V. Ozbolat , and I. T. Ozbolat , “3D Coaxial Bioprinting: Process Mechanisms, Bioinks and Applications,” Progress in Biomedical Engineering 4, no. 2 (2022): 022003, 10.1088/2516-1091/ac631c. 35573639 PMC9103990

[112] M. Askari , M. A. Naniz , M. Kouhi , A. Saberi , A. Zolfagharian , and M. Bodaghi , “Recent Progress in Extrusion 3D Bioprinting of Hydrogel Biomaterials for Tissue Regeneration: A Comprehensive Review With Focus on Advanced Fabrication Techniques,” Biomaterials science 9, no. 3 (2021): 535–573, 10.1039/D0BM00973C. 33185203

[113] J. W. Bae , J. H. Choi , Y. Lee , and K. D. Park , “Horseradish Peroxidase‐Catalysed in Situ‐Forming Hydrogels for Tissue‐Engineering Applications,” Journal of Tissue Engineering and Regenerative Medicine 9, no. 11 (2015): 1225–1232, 10.1002/term.1917. 24916126

[114] H. Sies and D. P. Jones , “Reactive Oxygen Species (ROS) as Pleiotropic Physiological Signalling Agents,” Nature Reviews Molecular Cell Biology 21, no. 7 (2020): 363–383, 10.1038/s41580-020-0230-3. 32231263

[115] A.‐E. Segneanu , L. E. Bejenaru , C. Bejenaru , et al., “Advancements in Hydrogels: A Comprehensive Review of Natural and Synthetic Innovations for Biomedical Applications,” Polymers 17, no. 15 (2025): 2026, 10.3390/polym17152026. 40808075 PMC12349326

[116] S. Naeimipour , F. Rasti Boroojeni , R. Selegård , and D. Aili , “Enzymatically Triggered Deprotection and Cross‐Linking of Thiolated Alginate‐Based Bioinks,” Chemistry of Materials 34, no. 21 (2022): 9536–9545, 10.1021/acs.chemmater.2c02037.

[117] L. S. M. Teixeira , J. Feijen , C. A. van Blitterswijk , P. J. Dijkstra , and M. Karperien , “Enzyme‐Catalyzed Crosslinkable Hydrogels: Emerging Strategies for Tissue Engineering,” Biomaterials 33, no. 5 (2012): 1281–1290, 10.1016/j.biomaterials.2011.10.067. 22118821

[118] B. Yin , M. Gosecka , M. Bodaghi , et al., “Engineering Multifunctional Dynamic Hydrogel for Biomedical and Tissue Regenerative Applications,” Chemical Engineering Journal 487 (2024): 150403, 10.1016/j.cej.2024.150403.

[119] N. Kumar , J. He , and J. F. Rusling , “Electrochemical Transformations Catalyzed by Cytochrome P450s and Peroxidases,” Chemical Society Reviews 52, no. 15 (2023): 5135–5171, 10.1039/D3CS00461A. 37458261

[120] C. Shen and Y. Wang , “Recent Progress on Peroxidase Modification and Application,” Applied Biochemistry and Biotechnology 196, no. 9 (2024): 5740–5764, 10.1007/s12010-023-04835-w. 38180646

[121] W. Mubarok , C. Zhang , and S. Sakai , “3D Bioprinting of Sugar Beet Pectin Through Horseradish Peroxidase‐Catalyzed Cross‐Linking,” ACS Applied Bio Materials 7, no. 5 (2024): 3506–3514, 10.1021/acsabm.4c00418. 38696441

[122] H. Pan , Y. Qu , F. Wang , S. Zhao , and G. Chen , “Horseradish Peroxidase‐Catalyzed Crosslinking Injectable Hydrogel for Bone Repair and Regeneration,” Colloid and Interface Science Communications 66 (2025): 100828.

[123] S. Naeimipour , F. R. Boroojeni , P. Lifwergren , R. Selegård , and D. Aili , “Multimodal and Dynamic Cross‐Linking of Modular Thiolated Alginate‐Based Bioinks,” Materials Today Advances 19 (2023): 100415, 10.1016/j.mtadv.2023.100415.

[124] A. Mirzahosseini and B. Noszál , “Species‐Specific Standard Redox Potential of Thiol‐Disulfide Systems: A Key Parameter to Develop Agents Against Oxidative Stress,” Scientific Reports 6, no. 1 (2016): 1–11, 10.1038/srep37596. 27869189 PMC5116634

[125] M. Patenaude , N. M. B. Smeets , and T. Hoare , “Designing Injectable, Covalently Cross‐Linked Hydrogels for Biomedical Applications,” Macromolecular Rapid Communications 35 (2014): 598–617, 10.1002/marc.201300818. 24477984

[126] O. Guillame‐Gentil , O. Semenov , A. S. Roca , et al., “Engineering the Extracellular Environment: Strategies for Building 2D and 3D Cellular Structures,” Advanced Materials 22, no. 48 (2010): 5443–5462, 10.1002/adma.201001747. 20842659

[127] H. Bi , X. Zhang , Q. Wang , et al., “Dynamic Reversible Disulfide Bonds Hydrogel of Thiolated Galactoglucomannan/Cellulose Nanofibril With Self‐Healing Property for Protein Release,” Industrial Crops and Products 206 (2023): 117615, 10.1016/j.indcrop.2023.117615.

[128] H.‐W. Chien , X. Xu , J.‐R. Ella‐Menye , W.‐B. Tsai , and S. Jiang , “High Viability of Cells Encapsulated in Degradable Poly (carboxybetaine) Hydrogels,” Langmuir 28, no. 51 (2012): 17778–17784, 10.1021/la303390j. 23163350

[129] S. S. Anumolu , A. R. Menjoge , M. Deshmukh , et al., “Doxycycline Hydrogels With Reversible Disulfide Crosslinks for Dermal Wound Healing of Mustard Injuries,” Biomaterials 32, no. 4 (2011): 1204–1217, 10.1016/j.biomaterials.2010.08.117. 20950853 PMC2995374

[130] F. El‐Mohtadi , R. Arcy , and N. Tirelli , “Oxidation‐Responsive Materials: Biological Rationale, State of the Art, Multiple Responsiveness, and Open Issues,” Macromolecular Rapid Communications 40, no. 1 (2019): 1800699, 10.1002/marc.201800699. 30474897

[131] D. Chimene , R. Kaunas , and A. K. Gaharwar , “Hydrogel Bioink Reinforcement for Additive Manufacturing: A Focused Review of Emerging Strategies,” Advanced Materials 32 (2020): 1902026, 10.1002/adma.201902026. 31599073

[132] P. Pal , S. Sambhakar , and S. Paliwal , “Revolutionizing Ophthalmic Care: A Review of Ocular Hydrogels From Pathologies to Therapeutic Applications,” Current Eye Research 50, no. 1 (2025): 1–17, 10.1080/02713683.2024.2396385. 39261982

[133] J. Kale , “Processed Excipients in Nasal Drug Delivery,” in Processed Excipients in Advanced Drug Delivery (CRC Press, 2025).

[134] V. G. Muir and J. A. Burdick , “Chemically Modified Biopolymers for the Formation of Biomedical Hydrogels,” Chemical Reviews 121, no. 18 (2020): 10908–10949, 10.1021/acs.chemrev.0c00923. 33356174 PMC8943712

[135] Y. Zhang , Z. Wang , Q. Sun , Q. Li , S. Li , and X. Li , “Dynamic Hydrogels With Viscoelasticity and Tunable Stiffness for the Regulation of Cell Behavior and Fate,” Materials 16, no. 14 (2023): 5161, 10.3390/ma16145161. 37512435 PMC10386333

[136] A. Sharma , A. Chand , I. Singh , and B. Gaur , “Vitrimers for 3D Printing Technology: Current Status and Future Perspectives,” Industrial & Engineering Chemistry Research 64, no. 5 (2025): 2491–2515, 10.1021/acs.iecr.4c03705.

[137] A. S. Finny , “3D Bioprinting in Bioremediation: A Comprehensive Review of Principles, Applications, and Future Directions,” PeerJ 12 (2024): 16897, 10.7717/peerj.16897. PMC10859081 38344299

[138] J. Elango and C. Zamora‐Ledezma , “Rheological, Structural, and Biological Trade‐Offs in Bioink Design for 3d Bioprinting,” Gels 11, no. 8 (2025): 659, 10.3390/gels11080659. 40868789 PMC12385449

[139] P. Casuso , I. Odriozola , A. Pérez‐San Vicente , et al., “Injectable and Self‐Healing Dynamic Hydrogels Based on Metal (I)‐thiolate/Disulfide Exchange as Biomaterials With Tunable Mechanical Properties,” Biomacromolecules 16, no. 11 (2015): 3552–3561, 10.1021/acs.biomac.5b00980. 26418440

[140] Y. Han , Y. Cao , and H. Lei , “Dynamic Covalent Hydrogels: Strong yet Dynamic,” Gels 8, no. 9 (2022): 577, 10.3390/gels8090577. 36135289 PMC9498565

[141] M. Benhar , “Oxidants, Antioxidants and Thiol Redox Switches in the Control of Regulated Cell Death Pathways,” Antioxidants 9, no. 4 (2020): 309, 10.3390/antiox9040309. 32290499 PMC7222211

[142] J. E. P. Brouns , (2024) “Engineering Function into Biomaterials Using Supramolecular Interactions: Turning Static Functions into Dynamic Reciprocity,” PhD thesis, Eindhoven University of Technology.

[143] T. I. Zarembinski , N. J. Doty , I. E. Erickson , R. Srinivas , B. M. Wirostko , and W. P. Tew , “Thiolated Hyaluronan‐Based Hydrogels Crosslinked Using Oxidized Glutathione: An Injectable Matrix Designed for Ophthalmic Applications,” Acta biomaterialia 10, no. 1 (2014): 94–103, 10.1016/j.actbio.2013.09.029. 24096152

[144] L. A. Pérez , R. Hernández , J. M. Alonso , R. Pérez‐González , and V. Sáez‐Martínez , “Hyaluronic Acid Hydrogels Crosslinked in Physiological Conditions: Synthesis and Biomedical Applications,” Biomedicines 9, no. 9 (2021): 1113. 34572298 10.3390/biomedicines9091113 PMC8466770

[145] M. P. Sekar , S. Suresh , A. Zennifer , S. Sethuraman , and D. Sundaramurthi , “Hyaluronic Acid as Bioink and Hydrogel Scaffolds for Tissue Engineering Applications,” ACS Biomaterials Science & Engineering 9, no. 6 (2023): 3134–3159, 10.1021/acsbiomaterials.3c00299. 37115515

[146] S. Tavakoli , N. Krishnan , H. Mokhtari , O. P. Oommen , and O. P. Varghese , “Fine‐Tuning Dynamic Cross–Linking for Enhanced 3D Bioprinting of Hyaluronic Acid Hydrogels,” Advanced Functional Materials 34, no. 4 (2024): 2307040, 10.1002/adfm.202307040.

[147] J. Liu , B. Yang , M. Li , J. Li , and Y. Wan , “Enhanced Dual Network Hydrogels Consisting of Thiolated Chitosan and Silk Fibroin for Cartilage Tissue Engineering,” Carbohydrate polymers 227 (2020): 115335. 31590851 10.1016/j.carbpol.2019.115335

[148] H. Zhang , A. Qadeer , and W. I. Chen , “In situ Gelable Interpenetrating Double Network Hydrogel Formulated From Binary Components: Thiolated Chitosan and Oxidized Dextran,” Biomacromolecules 12, no. 5 (2011): 1428–1437. 21410248 10.1021/bm101192b PMC3090471

[149] J. Yu , X. Xu , F. Yao , et al., “In Situ Covalently Cross‐Linked PEG Hydrogel for Ocular Drug Delivery Applications,” International Journal of Pharmaceutics 470, no. 1–2 (2014): 151–157, 10.1016/j.ijpharm.2014.04.053. 24768405

[150] C. Hiemstra , L. J. van der Aa , Z. Zhong , P. J. Dijkstra , and J. Feijen , “Novel In Situ Forming, Degradable Dextran Hydrogels by Michael Addition Chemistry: Synthesis, Rheology, and Degradation,” Macromolecules 40, no. 4 (2007): 1165–1173, 10.1021/ma062468d.

[151] C. Chen , A. Dong , and J. Yang , “Preparation and Properties of an Injectable Thermo‐Sensitive Double Crosslinking Hydrogel Based on Thiolated Chitosan/Beta‐Glycerophosphate,” Journal of Materials Science 47, no. 5 (2012): 2509–2517, 10.1007/s10853-011-6075-6.

[152] Z. Lin , R. Li , Y. Liu , et al., “Histatin1‐Modified Thiolated Chitosan Hydrogels Enhance Wound Healing by Accelerating Cell Adhesion, Migration and Angiogenesis,” Carbohydrate Polymers 230 (2020): 115710, 10.1016/j.carbpol.2019.115710. 31887922

[153] S. Asim , C. Tuftee , A. T. Qureshi , et al., “Multi‐Functional Gelatin‐Dithiolane Hydrogels for Tissue Engineering,” Advanced Functional Materials 35, no. 3 (2025): 2407522, 10.1002/adfm.202407522.

[154] C. Zhao , Z. Wu , H. Chu , et al., “Thiol‐Rich Multifunctional Macromolecular Crosslinker for Gelatin‐Norbornene‐Based Bioprinting,” Biomacromolecules 22, no. 6 (2021): 2729–2739, 10.1021/acs.biomac.1c00421. 34057830

[155] S. Tavakoli , A. Kocatürkmen , O. P. Oommen , and O. P. Varghese , “Ultra‐Fine 3D Bioprinting of Dynamic Hyaluronic Acid Hydrogel for In Vitro Modeling,” Advanced Materials 37 (2025): 2500315, 10.1002/adma.202500315. 40357760 PMC12306391

[156] J. Zhang , Y. Zeng , Y. Heng , et al., “Enzyme‐Assisted Activation Technique for Producing Versatile Hydrogel Microparticle Scaffolds With High Surface Chemical Reactivity,” Advanced Functional Materials 34, no. 30 (2024): 2400858, 10.1002/adfm.202400858.

[157] Y. Wu , D. J. Ravnic , and I. T. Ozbolat , “Intraoperative Bioprinting: Repairing Tissues and Organs in a Surgical Setting,” Trends in biotechnology 38, no. 6 (2020): 594–605, 10.1016/j.tibtech.2020.01.004. 32407688 PMC7666846

[158] Z. Mahmoudi , M. Sedighi , A. Jafari , et al., “In Situ 3D Bioprinting: A Promising Technique in Advanced Biofabrication Strategies,” Bioprinting 31 (2023): 00260.

[159] J. Han , J. Park , R. Bhatta , et al., “A Double Crosslinking Adhesion Mechanism for Developing Tough Hydrogel Adhesives,” Acta Biomaterialia 150 (2022): 199–210, 10.1016/j.actbio.2022.07.028. 35870776

[160] E. Gangadevi , M. L. Shri , R. K. Dhanaraj , and B. Balusamy , Computational Intelligence in Bioprinting: Challenges and Future Directions (John Wiley & Sons, 2024), 10.1002/9781394204878.

[161] S. B. Majee , S. Singh , and U. Das , “Future Directions and Emerging Trends in 3D Printing Integrating With Artificial Intelligence and Machine Learning,” in Precision 3D Printing in Pharmaceutical Sciences: A Transformative Shift in Drug Manufacturing and Delivery Systems (Scrivener Publishing LLC, 2025), 443–488, 10.1002/9781394337576.

[162] Z. Zhang , X. Zhou , Y. Fang , Z. Xiong , and T. Zhang , “AI‐Driven 3D Bioprinting for Regenerative Medicine: From Bench to Bedside,” Bioactive Materials 45 (2025): 201–230, 10.1016/j.bioactmat.2024.11.021. 39651398 PMC11625302

